# Very high sensitivity of African rice to artificial ultraviolet-B radiation caused by genotype and quantity of cyclobutane pyrimidine dimer photolyase

**DOI:** 10.1038/s41598-020-59720-x

**Published:** 2020-02-21

**Authors:** Gideon Sadikiel Mmbando, Mika Teranishi, Jun Hidema

**Affiliations:** Graduate School of Life Sciences, Tohoku University, Sendai, 980-8577 Japan

**Keywords:** Light stress, Biodiversity

## Abstract

Ultraviolet-B (UVB) radiation damages plants and decreases their growth and productivity. We previously demonstrated that UVB sensitivity varies widely among Asian rice (*Oryza sativa* L.) cultivars and that the activity of cyclobutane pyrimidine dimer (CPD) photolyase, which repairs UVB-induced CPDs, determines UVB sensitivity. Unlike Asian rice, African rice (*Oryza glaberrima* Steud. and *Oryza barthii* A. Chev.) has mechanisms to adapt to African climates and to protect itself against biotic and abiotic stresses. However, information about the UVB sensitivity of African rice species is largely absent. We showed that most of the African rice cultivars examined in this study were UVB-hypersensitive or even UVB-super-hypersensitive in comparison with the UVB sensitivity of Asian *O. sativa* cultivars. The difference in UVB resistance correlated with the total CPD photolyase activity, which was determined by its activity and its cellular content. The UVB-super-hypersensitive cultivars had low enzyme activity caused by newly identified polymorphisms and low cellular CPD photolyase contents. The new polymorphisms were only found in cultivars from West Africa, particularly in those from countries believed to be centres of *O. glaberrima* domestication. This study provides new tools for improving both Asian and African rice productivity.

## Introduction

Plants use sunlight for photosynthesis and are therefore exposed to ultraviolet-B (UVB) radiation (280–315 nm). Damage caused by UVB radiation decreases plant growth and productivity^1^. Artificial UVB radiation in a growth chamber or field can also damage plants, decreasing the growth and productivity of economically important crops, including rice; UV radiation exclusion prevents such damage and can increase plant growth^2,3^.

Rice is one of the most important staple grains globally and is extensively cultivated worldwide in regions with different climates. The genus *Oryza* comprises 22 wild species and 2 species of cultivated rice (*Oryza sativa* L. and *O. glaberrima* Steud.); *O. sativa* and *O. glaberrima* originated from and were domesticated in Asia and West Africa, respectively^4,5^. Asian rice cultivars belong to one of the two major *O. sativa* subspecies, *japonica* or *indica*. UVB sensitivity varies widely among Asian rice cultivars^6^ due to differences in the enzymatic activity for repair of UV-induced DNA damage^7^. Upon UVB irradiation, cyclobutane pyrimidine dimers (CPDs) are formed between adjacent pyrimidines on the same DNA strand^8^. In the photoreactivation pathway, the enzyme photolyase absorbs light in the UVA (315–400 nm) and blue ranges through the FAD chromophore, which releases energy to induce dimer dissociation into monomers^9^. Photoreactivation activity is higher in the UVB-resistant rice cultivar Sasanishiki (*O. sativa* ssp. *japonica*) than in the less resistant cultivar Norin 1 (also *japonica*)^10^. The higher activity in Sasanishiki results from spontaneous mutations in the CPD photolyase gene that alter the function of the enzyme rather than from a regulatory mutation^11^. The *indica* rice cultivar Surjamkhi is even more sensitive (hypersensitive) to UVB than UVB-sensitive Norin 1 and has lower photoreactivation activity because of changes in the deduced amino acid sequence of CPD photolyase in comparison with that of Norin 1^12^.

The origin of African rice (*O. glaberrima* and *Oryza barthii* A. Chev.) differs from that of Asian rice (*O. sativa*), although some *O. sativa* has been introduced and adapted to African climates^13^. *O. glaberrima* is well adapted for cultivation in West Africa and is tolerant to biotic and abiotic stresses such as drought, soil acidity, and iron and aluminium toxicity^14–16^. Therefore, one might expect cultivars from tropical Africa to be resistant to UVB radiation stress. However, information about UVB resistance and CPD photolyase genotypes among African rice cultivars (*O. sativa, O. barthii* and *O. glaberrima*) is largely absent, although a high-quality assembly and annotation of the *O. glaberrima* genome has been reported^5^.

In this study, we investigated the UVB resistance and CPD photolyase activity and content in 15 African rice cultivars grown in different regions in Africa and belonging to *O. sativa, O. barthii* or *O. glaberrima*. Unexpectedly, we found that most *O. glaberrima* and *O. barthii* cultivars are more sensitive to UVB than an Asian hypersensitive *O. sativa* cultivar (Surjamkhi) under laboratory conditions at a specific UVB intensity (UVB_BE_: 14.7 kJ/m^2^/d) and that African rice has a low CPD photolyase activity and cellular content; these characteristics are associated with a new CPD photolyase genotype.

## Results

### African rice cultivars have a UVB-hypersensitive phenotype

First, we investigated the effects of UVB radiation on the growth of 15 African rice cultivars belonging to tropical *O. sativa* (TOS) 7940, 8086, 8722, 10589, 13649, 13699 and 14844, tropical *O. barthii* (TOB) 7307 and 14466, and tropical *O. glaberrima* (TOG) 12380, 14928, Jiakawo Wodewo, MB3, C7251 and Maro Goudo, which are mainly grown in different regions of Africa.

**Figure 1:**
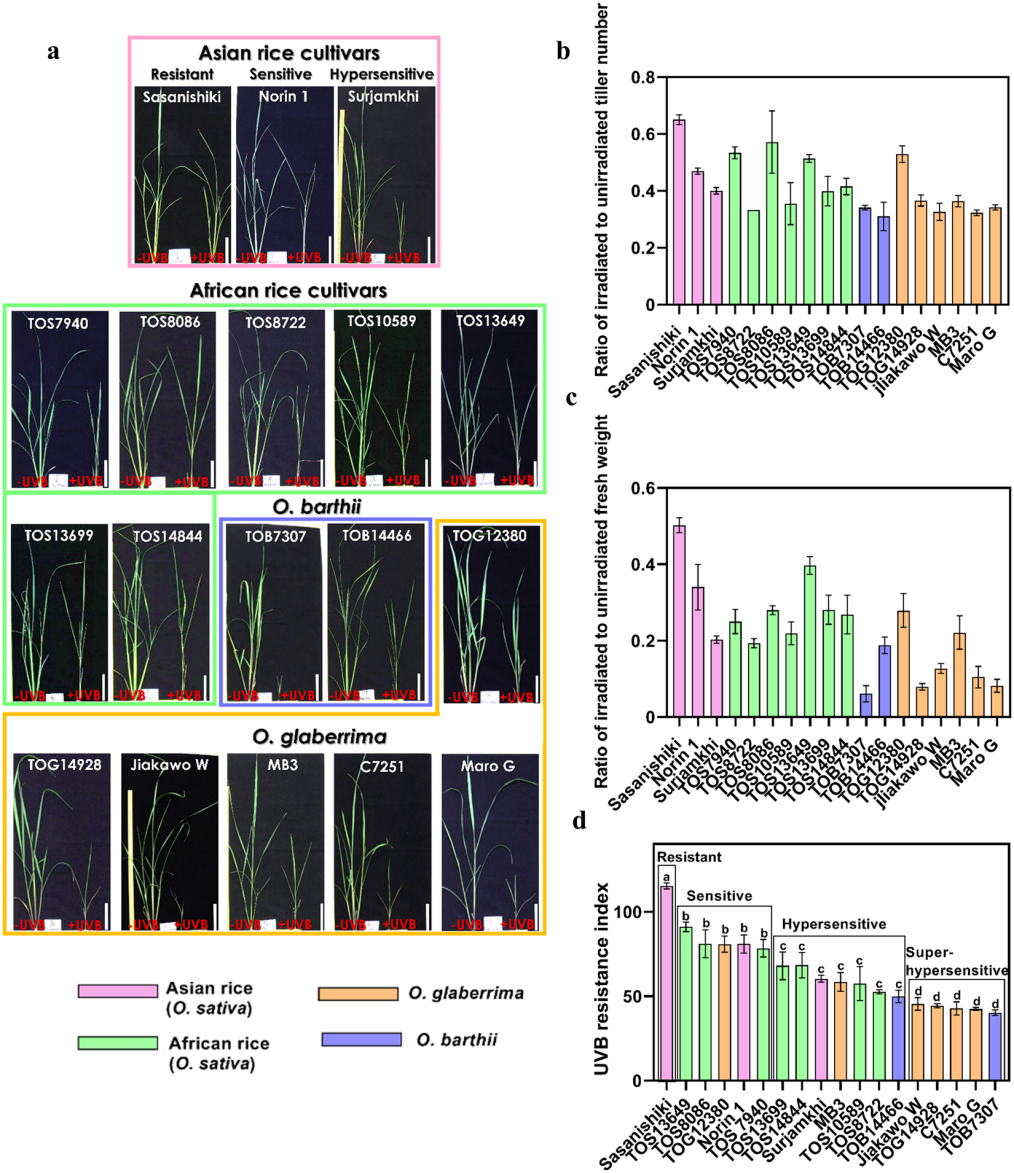
African rice cultivars are highly susceptible to damage by UVB radiation stress. (**a**) Plants of three rice species (*Oryza sativa*, *O. barthii* and *O. glaberrima*) were grown in a growth cabinet for 21 days with (+UVB) or without (−UVB) UVB radiation. Maro G, Maro Goudo; Jiakawo W, Jiakawo Wodewo. UVB resistance varied widely among African rice cultivars. Bars = 5 cm. (**b**) Ratio of irradiated to unirradiated tiller number (+UVB)/(−UVB). (**c**) Ratio of irradiated to unirradiated above-ground fresh weight. (**d**) UVB resistance index was determined by summing the value of the ratio of +UVB to −UVB of tiller number and above-ground fresh weight × 100, i.e., (+UVB)/(−UVB) × 100. Values are means ± SD based on three independent experimental replicates (n = 9), each performed in triplicate in (**b**–**d**); different letters indicate significant differences determined by the Tukey-Kramer test (*P < *0.05).

We previously reported that UVB sensitivity varies widely among Asian rice cultivars (*O. sativa*, ssp. *japonica* and *indica*), which are classified as UVB resistant, UVB sensitive or UVB hypersensitive^7^. These phenotypes were clearly observed when rice plants are grown under visible radiation supplemented with UVB radiation in a growth chamber^7^. Under visible radiation alone, the plant shape and above-ground fresh weight varied among cultivars, although there was no significant difference in tiller number (Figs. 1a and S1). The rice plants were grown under visible radiation with supplemented UVB (biologically effective UVB radiation, UVB_BE_: 14.7 kJ/m^2^/d), the same intensity as used in a previous study^17^, and the growth of all cultivars was suppressed; the degree of suppression differed considerably among cultivars of the same species (Fig. 1a). The effects of UVB treatment on the tiller number and above-ground fresh weight were investigated. Here, the values of the middle three of five plants were used for calculating the following score because poorly performing plants appeared occasionally regardless of UVB treatment. In African rice, the ratio of irradiated to unirradiated tiller number ranged from 0.32 in TOB14466, C7251 and Jiakawo Wodewo to 0.58 in TOS8086, and the ratio of irradiated to unirradiated above-ground fresh weight ranged from 0.06 in TOB7307 to 0.40 in TOS13649 (Fig. 1b,c and Table S1). We determined the UVB resistance index by multiplying the sum of the values of the ratio of irradiated to unirradiated tiller number and above-ground fresh weight by 100^18^. In African rice, the UVB resistance index ranged from 92 (least affected; TOS13649) to 40 (most affected; TOB7307) (Fig. 1d and Table S1). UVB resistance varied widely among the African cultivars, similar to Asian rice cultivars^19^, and all African cultivars were more sensitive than the UVB-resistant *japonica* cultivar Sasanishiki (UVB resistance index of Sasanishiki: 117) (Fig. 1d and Table S1). Moreover, the UVB-sensitive phenotypes of some African cultivars were more pronounced than those of the UVB-hypersensitive *indica* cultivar Surjamkhi. On the basis of significant differences between UVB resistance indexes (determined with the Tukey-Kramer test), we classified rice cultivars into four groups: (1) UVB-resistant, (2) UVB-sensitive, (3) UVB-hypersensitive and (4) UVB-super-hypersensitive (Fig. 1d); the last group was newly identified in this study. Some *O. glaberrima* and *O. barthii* were classified as UVB-super-hypersensitive, and other African cultivars were classified as either hypersensitive or sensitive (Fig. 1d). No African cultivars were classified as UVB resistant.

### Various CPD photolyase genotypes exist in African rice

**Figure 2:**
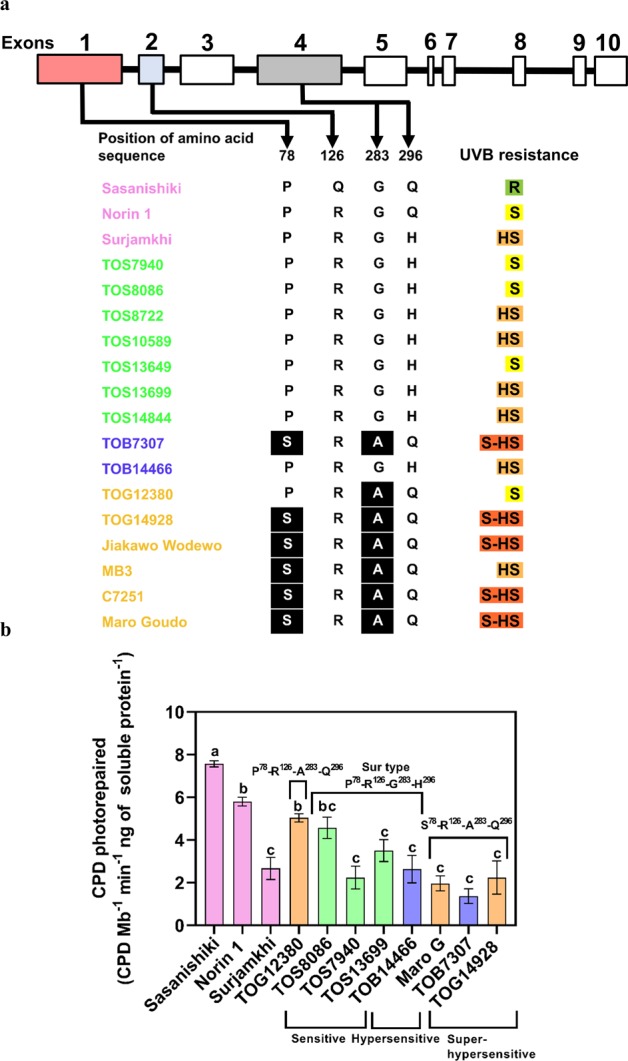
S^78^ and A^283^ polymorphisms in cyclobutane pyrimidine dimer (CPD) photolyase affect CPD photorepair activity. (**a**) CPD photolyase genotypes. P^78^-R^126^-G^283^-H^296^, P^78^-R^126^-A^283^-Q^296^ and S^78^-R^126^-A^283^-Q^296^ were found in African rice. Amino acid residues highlighted in black (S^78^ and A^283^) were mainly found in African rice cultivars that are UVB-super-hypersensitive in comparison with Sasanishiki. UVB resistance: R = resistance, S = sensitive, HS = hypersensitive and S-HS = super-hypersensitive. (**b**) CPD photorepair activity in crude soluble protein of third fully expanded leaves. Leaf extracts were mixed with UV-irradiated λDNA and exposed to continuous blue light for 6 h. Activity varied widely among African rice species and tended to be lowest in UVB-super-hypersensitive cultivars. Values are means ± SD. n = 3 replicates in **(b**); different letters indicate significant differences determined by the Tukey-Kramer test (*P < *0.05). Colour coding is as in Fig. 1.

The differences in UVB sensitivity among Asian rice cultivars were determined according to the genotype of CPD photolyase^12,19^. The deduced amino acid sequence of CPD photolyase in UVB-resistant Sasanishiki at positions 126 and 296 was Q^126^-Q^296^, “Sasa-type”, whereas that in UVB-sensitive Norin 1 was R^126^-Q^296^, “Nori-type”, and that in UVB-hypersensitive Surjamkhi was R^126^-H^296^, “Sur-type”. These differences affected the activity of CPD photolyase^9^. We therefore determined the sequences of the CPD photolyase genes in the African rice cultivars. All CPD photolyase genes sequenced in this study had 10 exons, 9 introns and a 1518-bp ORF encoding a 506-amino-acid protein (Figs. 2a, S2 and S3). All cultivars examined had R^126^ (as in the Nori- and Sur-types). Q^296^ (as in the Sasa- and Nori-types) was found in TOB7307, TOG12380, TOG14928, Jiakawo Wodewo, MB3, C7251, and Maro Goudo, whereas H^296^ (as in the Sur-type) was found in all African *O. sativa* (TOS) cultivars examined and in TOB14466 (Fig. 2a). In addition to these differences from the Sasa-type, we found novel amino acid substitutions in the African cultivars that had Q^296^. The cytosine at position 232 in exon 1 in Sasanishiki was changed to thymine in the *O. glaberrima* cultivars C7251, Jiakawo Wodewo, Maro Goudo, TOG14928 and MB3 and in TOB7307 (*O. barthii*), leading to an amino acid change at position 78 from P to S (Figs. 2a, S2 and S3). The guanine at position 848 in exon 4 in Sasanishiki was changed to cytosine in the same cultivars and in TOG12380, leading to an amino acid change at position 283 from G to A (Figs. 2a, S2 and S3). Therefore, these cultivars (except TOG12380) had S^78^-R^126^-A^283^-Q^296^, and TOG12380 had P^78^-R^126^-A^283^-Q^296^. None of the African rice cultivars examined had the Sasa-type CPD photolyase. Interestingly, all African cultivars with S^78^-R^126^-A^283^-Q^296^ except MB3 were UVB-super-hypersensitive (Fig. 1d). Our results revealed that S^78^-R^126^-A^283^-Q^296^ is widely conserved among UVB-super-hypersensitive African rice cultivars with different genetic and geographical backgrounds.

**Figure 3:**
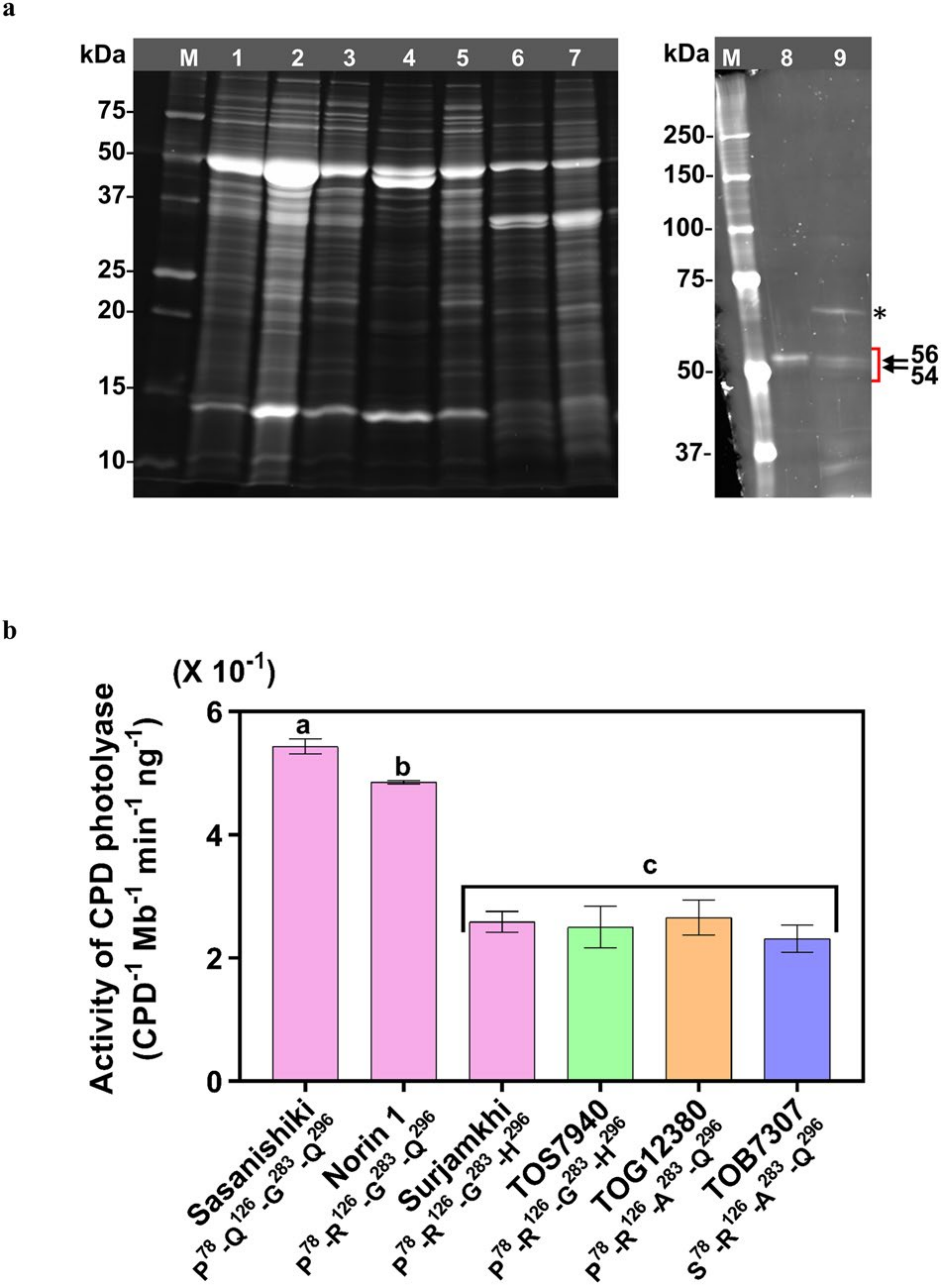
A^283^ but not S^78^ reduces the activity of CPD photolyase purified from African rice species. (**a**) whole plants were used for purification of native CPD photolyase protein from the African rice cultivar TOS7940. Electrophoresis was performed in a 12.5% or 7.5% SDS-polyacrylamide gel, which was stained with SYPRO Ruby stain. Lane 1, crude extract (fraction 1); lane 2, protein precipitated with ammonium sulphate between 35% and 70% saturation (fraction 2); lane 3, flow-through fraction from a UNO-Q12 anion-exchange column (fraction 3); lane 4, protein eluted from the UNO-Q12 column; lane 5, flow-through from a heparin affinity column; lane 6, protein eluted from the heparin column (fraction 4); lane 7, protein not bound to UV-irradiated DNA-conjugated magnetic beads; lanes 8 and 9, protein purified with UV-irradiated DNA-conjugated magnetic beads (fraction 5) of cultivars S-C (Sasa-type-CPD photolyase overexpressing transgenic rice plant) and TOS7940, respectively; M, molecular weight marker. Arrows indicate phosphorylated (56 kDa) and unphosphorylated (54 kDa) CPD photolyase. The asterisk indicates that BSA was detected in our final purification fraction. (**b**) CPD photorepair activity of purified native CPD photolyase. African cultivars (TOS7940, TOG12380 and TOB7307) and Surjamkhi showed significantly lower activity of CPD photolyase than that of Sasanishiki and Norin 1. Values are the mean ± SD. N = 3 replicates in **c**.; different letters indicate significant differences determined by the Tukey-Kramer test (*P* < 0.05); c, not significant. Colour coding is as in Fig. 1.

### Lower CPD photorepair activity was observed *in vitro* in UVB-super-hypersensitive African rice cultivars

We hypothesized that the S^78^-R^126^-A^283^-Q^296^ genotype decreases CPD photorepair activity. To examine this possibility, we measured CPD photorepair activity in crude extracts of the third leaves of representative African cultivars using UV-irradiated λDNA as a substrate (Fig. 2b)^11^. The CPD photorepair activities in the UVB-super-hypersensitive cultivars TOG14928, TOB7307 and Maro Goudo (S^78^-R^126^-A^283^-Q^296^) were similar to those of the Sur-type cultivars (P^78^-R^126^-G^283^-H^296^) but were significantly lower than those of Sasanishiki (P^78^-Q^126^-G^283^-Q^296^) and Norin 1 (P^78^-R^126^-G^283^-Q^296^). The activity of TOG12380 (P^78^-R^126^-A^283^-Q^296^) was highest among the African cultivars examined and similar to that in UVB-sensitive Norin 1 (P^78^-R^126^-G^283^-Q^296^) (Fig. 2b). These results indicate that the replacement of P^78^ with S and/or of G^283^ with A decreases the activity, possibly due to structural changes in CPD photolyase. On the other hand, the CPD photorepair activities varied among the Sur-type (P^78^-R^126^-G^283^-H^296^) cultivars, suggesting that other factors, such as CPD photolyase protein content, affect CPD photolyase activity.

### Decreased activity of CPD photolyase and protein content were observed in African rice cultivars

To confirm that S^78^ and/or A^283^ decrease the activity of CPD photolyase, we purified native CPD photolyase from leaves of the African cultivars TOB7307 (S^78^-R^126^-A^283^-Q^296^), TOG12380 (P^78^-R^126^-A^283^-Q^296^) and Sur-type TOS7940 (P^78^-R^126^-G^283^-H^296^) and three Asian cultivars according to our previous report^20^ (Figs. 3a and S4). Two forms of CPD photolyase, 54 kDa and 56 kDa (phosphorylated form), were detected by SDS-PAGE in the final purified fraction of each cultivar. However, the final purification fraction was not homogeneous due to the presence of contaminant bands (Figs. 3a and S4a). We found that one of the contaminant bands was that of BSA (66.5 kDa) by Matrix-Assisted Laser Desorption/Ionization-Time Of Flight (MALDI-TOF) analysis. In the final purification step, magnetic beads and all tubes were coated with 0.1 mg/ml BSA to inhibit the loss of purified protein due to surface absorption. BSA contamination occurred in almost all fractions of all cultivars examined here (Figs. 3a and S4). The protein amount of CPD photolyase of each cultivar was measured by the sum of the intensity of both of the 54 and 56 kDa bands by comparing the intensity of BSA as a standard protein. The CPD photolyase purity was ca. 53,000–165,000 times that in the crude extract (Table S2). The photorepair activity of the final purification fraction was measured, and then the photorepair activity was divided by the protein amount of CPD photolyase in the fraction to determine the activity of CPD photolyase for each cultivar (CPD/Mb/min/ng). The activity of CPD photolyase was significantly lower in all three African rice cultivars than in the UVB-resistant Sasanishiki or UVB-sensitive Norin 1 rice cultivars (Fig. 3b); the CPD photolyase activity was similar among the African cultivars and did not significantly differ between them and Surjamkhi. These results indicate that the replacement of P^78^ with S could not affect the activity of CPD photolyase when comparing that of TOG12380 (P^78^-R^126^-A^283^-Q^296^) with that of TOB7307 (S^78^-R^126^-A^283^-Q^296^). By contrast, when comparing the activity between Norin 1 (P^78^-R^126^-G^283^-Q^296^) and TOG12380 (P^78^-R^126^-A^283^-Q^296^), the replacement of G^283^ with A significantly reduced the activity of CPD photolyase to a similar extent as that of the replacement of Q^296^ with H. On the other hand, as described above (Fig. 2b), the activity in crude extracts of TOS8086 (Sur-type) and TOG12380 (P^78^-R^126^-A^283^-Q^296^) was higher than that of the other African cultivars (Sur-type or S^78^-R^126^-A^283^-Q^296^). The possible explanation could be that the CPD photolyase protein content in the crude extracts of TOS8086 and TOG12380 was higher than in the crude extracts of the other African cultivars; this difference was confirmed by estimation of the CPD photolyase protein content as a ratio of activity in the crude extracts to activity in purified CPD photolyase (Fig. S5). To test whether the differences in CPD photolyase content among cultivars resulted from the differences in CPD photolyase gene expression levels, we determined the levels of the CPD photolyase mRNA (Fig. S6a). The CPD photolyase gene expression levels varied widely among the African cultivars examined here. Although the CPD photolyase mRNA levels in most African rice cultivars, except TOS7940, TOS13649, TOS14844 and TOG12380, were higher than those in Surjamkhi, overall, there was no correlation between photolyase content and photolyase gene expression level (Fig. S6b). Thus, the differences in the CPD photolyase content might be caused by differences not only in gene transcription but also in protein stability.

### Decreased CPD binding to P^78^-R^126^-A^283^-Q^296^ and S^78^-R^126^-A^283^-Q^296^ CPD photolyase was observed

**Figure 4:**
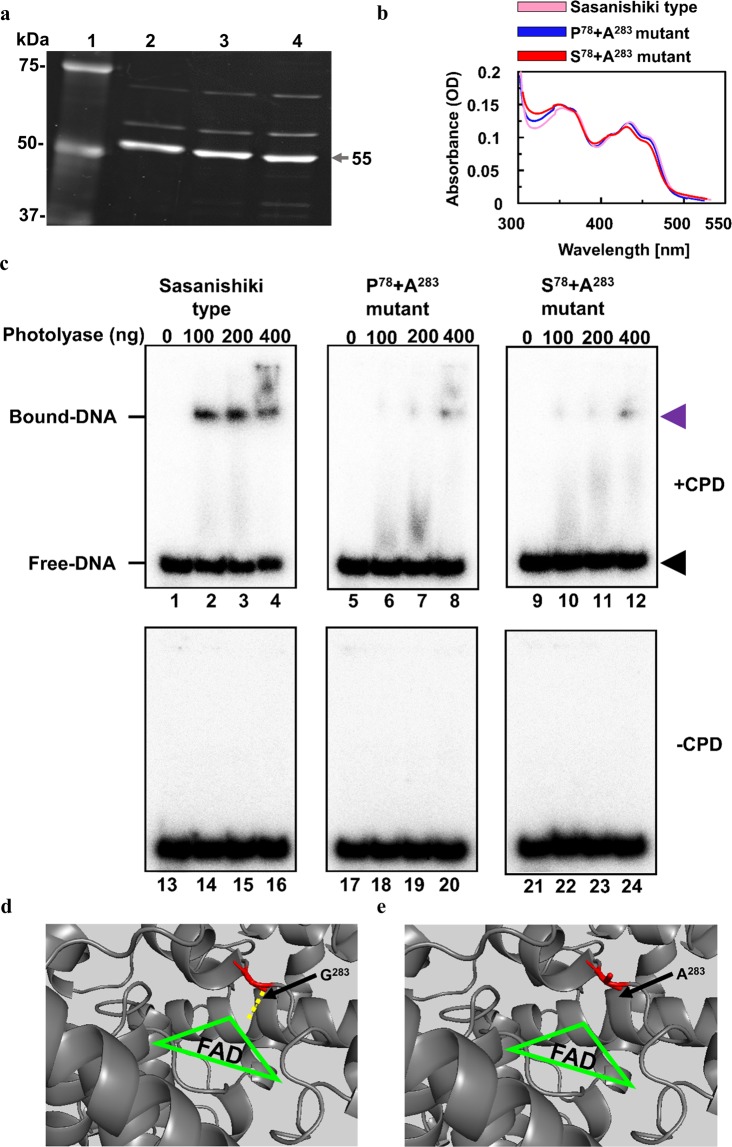
S^78^ and A^283^ decrease the binding of recombinant CPD photolyase to CPDs. (**a**) lane 1, molecular weight marker; lane 2, Sasanishiki-type CPD photolyase (P^78^-Q^126^-G^283^-Q^296^); lane 3, P^78^-R^126^-A^283^-Q^296^; lane 4, S^78^-R^126^-A^283^-Q^296^. Arrow, 55-kDa photolyase. (**b**) absorbance spectra of the photolyases shown in (**a**). There were no major differences in absorbance spectra among the CPD photolyase variants. (**c**) electrophoretic mobility shift assay to detect CPD photolyase binding to CPDs. Duplex DNA containing (lanes 1–12) or not containing (lanes 13–24) CPDs was incubated with increasing amounts of *E. coli*-expressed CPD photolyase variants. The P^78^ + A^283^ and S^78^ + A^283^ mutations reduced CPD photolyase binding to CPDs in comparison with the Sasanishiki type. Electrophoresis was performed in a 10% nondenaturing polyacrylamide gel. Purple arrowhead, bound DNA; black arrowhead, free DNA. (**d**,**e**) 3D structure of Sasa-type CPD photolyase containing glycine (G) at amino acid 283 (**d**) and TOB7307 CPD photolyase containing alanine (A) at amino acid 283 (**e**). Visualization of the rice CPD photolyase 3D structure was analysed by PyMol 2.2.2 software using amino sequence information of the selected cultivar TOB7307 compared to that of Sasanishiki (protein data bank code: 3UMV)^23^. Green triangle, FAD chromophores. Yellow dotted line, polar contact of amino acid position 283.

Using an electrophoretic mobility shift assay with *E. coli*-expressed CPD photolyase, we previously demonstrated that the relatively low activity of Sur-type CPD photolyase resulted from decreased CPD binding caused by replacement of Q^296^ in Sasa- or Nori-type CPD photolyases to the H residue^21^. We therefore tested whether the relatively low activity of P^78^-R^126^-A^283^-Q^296^ and S^78^-R^126^-A^283^-Q^296^ CPD photolyases resulted from decreased CPD binding caused by the replacement of G^283^ with A. Recombinant CPD photolyases (P^78^-R^126^-A^283^-Q^296^ or S^78^-R^126^-A^283^-Q^296^) purified from *E. coli* (Fig. 4a) were >80% pure. The absorption spectrum of each recombinant protein contained peaks at approximately 375 and 450 nm, which are unique to the FAD chromophore, with no major difference among the different proteins (Fig. 4b). These purified recombinant proteins were used in an electrophoretic mobility shift assay. No band shifts were detected with an oligonucleotide containing no CPDs (Fig. 4c). In the presence of an oligonucleotide containing CPDs, the intensity of the shifted band increased with increasing amounts of all recombinant proteins but was weaker with P^78^-R^126^-A^283^-Q^296^ and S^78^-R^126^-A^283^-Q^296^ CPD photolyases than with the Sasa-type photolyase (Fig. 4c). These results strongly indicate that the relatively low activity of P^78^-R^126^-A^283^-Q^296^ and S^78^-R^126^-A^283^-Q^296^ CPD photolyases resulted from decreased CPD binding caused by replacement of G^283^ with A.

To predict whether the replacement of P^78^ with S or that of G^283^ with A affects CPD photolyase structure, which would result in low CPD photorepair and binding activity, we visualized the rice CPD photolyase 3D structure with PyMol software based on our previous data^22^. The amino acid replacement of P with S at position 78 located close to the N terminus of CPD photolyase was predicted not to affect the hydrogen bond on the 3D structure, although the replacement of the nonpolar amino acid P with the aromatic and polar uncharged S seemed to change the structure (Fig. S7). In contrast, the replacement of G^283^ with A was predicted to cause structural changes through the loss of polar contacts between rice CPD photolyase and the FAD chromophore in the UVB-super-hypersensitive cultivar TOB7307 (Fig. 4d,e). G^283^ of rice CPD photolyase is directly bound to FAD by hydrogen bonding^22^. The FAD binding site is closely located to the substrate CPD binding pocket (Fig. 7S)^22^. Therefore, the structural change that occurred adjacent to the CPD binding pocket by the replacement of G with A at position 283 might affect the binding activity of CPD (Fig. 4c).

### UVB resistance strongly depends on total leaf CPD photolyase activity

**Figure 5:**
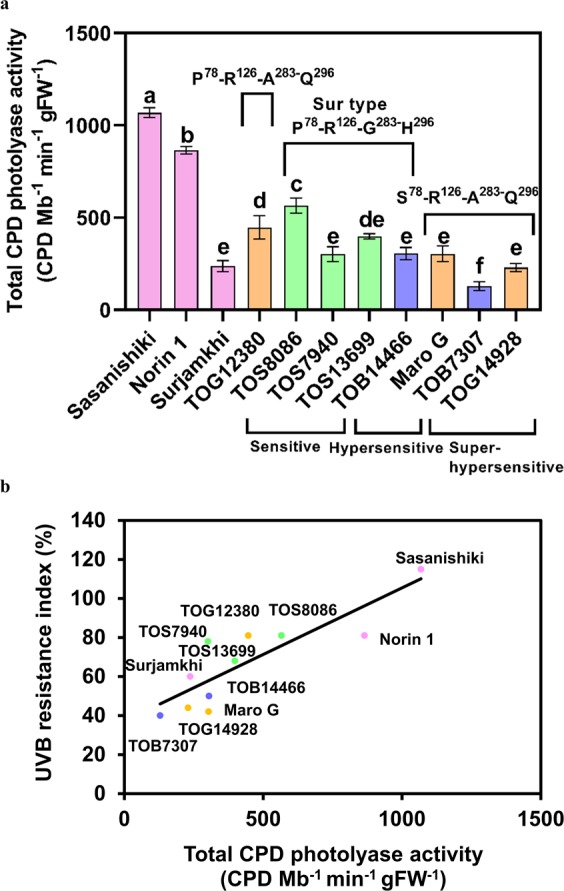
The UVB resistance index is strongly correlated with the total activity of CPD photolyase in the leaf. (**a**) total CPD photolyase activity was calculated from the CPD photolyase protein content [(pg of CPD photolyase)/(µg of soluble protein)], total soluble protein content (µg/gFW) and activity of CPD photolyase (CPD/Mb/min/ng). UVB-super-hypersensitive African rice cultivars have low total activity of CPD photolyase. (**b**) correlation between UVB resistance index (Fig. 1d) and total CPD photolyase activity shown in (**a**). Linear regression analysis showed a significant correlation between the data sets (*P < *0.01; *R*^*2*^ = 0.74). Values are means ± SD. n = 3 replicates in (**b**); different letters indicate significant differences determined by the Tukey-Kramer test (*P < *0.05). Colour coding is as in Fig. 1.

We previously found a strong correlation between UVB sensitivity and *in vitro* CPD photolyase activity or CPD photolyase genotype among Asian rice cultivars because the activity of CPD photolyase depends on its genotype^19^. However, UVB resistance among African rice cultivars did not depend on CPD photolyase activity or genotype. Therefore, the differences in UVB resistance might have been caused by the difference in the cell or leaf CPD photolyase content. To examine this possibility, we estimated the total CPD photolyase activity in a leaf (CPD/Mb/min/gFW or CPD/Mb/min/cm^2^) by multiplying the CPD photolyase activity in the crude extract (CPD/Mb/min/ng) and the soluble protein content per leaf fresh weight (mg/gFW) or per leaf area (mg/cm^2^). The soluble protein content per gram fresh weight or per leaf area varied among the African cultivars (Fig. S8), and the total CPD photolyase activity (CPD/Mb/min/gFW) varied widely among cultivars, even those with the same CPD photolyase genotypes (Fig. 5a). The lowest total CPD photolyase activity was found in the UVB-super-hypersensitive cultivar TOB7307 (S^78^-R^126^-A^283^-Q^296^), and the highest total CPD photolyase activity was found in the UVB-sensitive cultivar TOS8086 (P^78^-R^126^-G^283^-H^296^; Sur-type). A similar tendency was observed in total CPD photolyase activity based on leaf area (CPD/Mb/min/cm^2^) (Fig. S9a). To determine whether the differences in UVB resistance were caused by different total leaf CPD photolyase activities, we assessed the relationship between the UVB resistance index and total CPD photolyase activity and found a correlation in gFW (*P* < 0.01 for a linear model, *R*^2^ = 0.74; Fig. 5b) or cm^2^ (*P* < 0.01 for a linear model, *R*^2^ = 0.72; S9b). Our results therefore suggest that the UVB super-hypersensitivity of African rice cultivars is caused by low total CPD photolyase activity due to the low activity and content of CPD photolyase in the cell. Our findings also reveal that the differences in the UVB resistance index (Fig. 1d) or CPD photorepair activity in the crude extract (Fig. 2b) of cultivars with the same CPD photolyase genotype were caused mainly by differences in total CPD photolyase content.

### Origin of CPD photolyase genotypes in African rice

**Figure 6:**
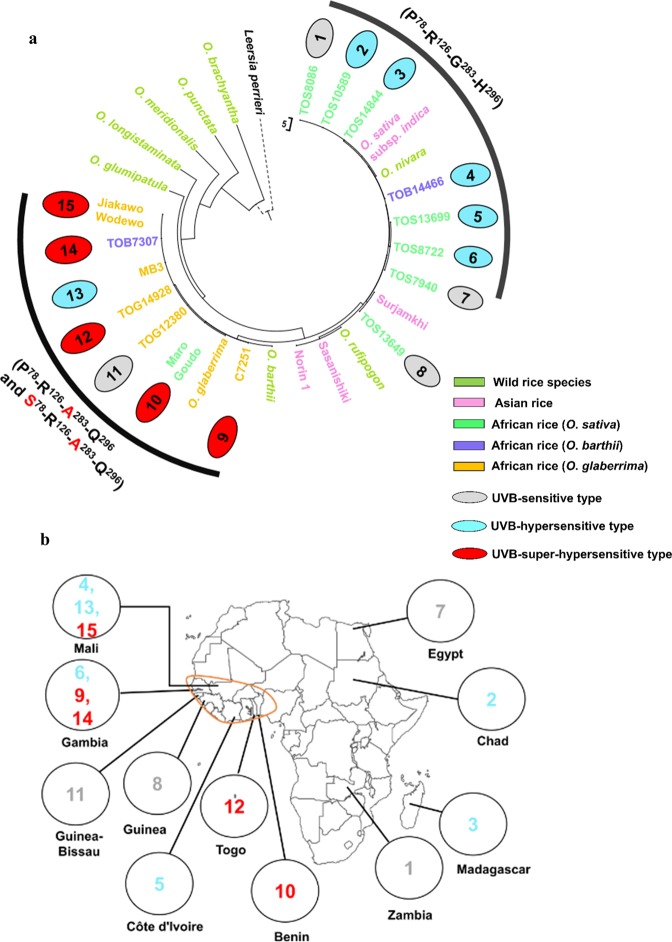
Evolution of the CPD photolyase gene in African rice. (**a**) UVB-super-hypersensitive African rice cultivars (S^78^-R^126^-A^283^-Q^296^) share the same clade with that of native African rice, *O. glaberrima* and *O. sativa* (Maro G). A phylogenetic tree was inferred using the UPGMA method^46^. (**b**) countries of origin of the 15 African cultivars (*O. sativa*, *O. barthii* and *O. glaberrima*) characterized in this study. UVB-super-hypersensitive African cultivars (S^78^ and A^283^) originated only from the area circled in orange, which was proposed to be the centre of *O. glaberrima* domestication.

We detected three CPD photolyase genotypes in African rice cultivars: P^78^-R^126^-G^283^-H^296^, which was the same as in the *indica* cultivar Surjamkhi; P^78^-R^126^-A^283^-Q^296^, found in *O. glaberrima*; and S^78^-R^126^-A^283^-Q^296^, which was present in *O. glaberrima* and *O. barthii*. To investigate the generation of these polymorphisms, we performed phylogenetic analysis of CPD photolyase genomic sequences from African, Asian and wild rice species from databases, including *O. brachyantha*, which has an F genome and belongs to a basal lineage in *Oryza*; its genome is therefore considered to be closely related to the ancestral *Oryza* genomes^23,24^. We also included the African species *Leersia perrieri* as an outgroup (Fig. 6a). Interestingly, neither P^78^-R^126^-A^283^-Q^296^ nor S^78^-R^126^-A^283^-Q^296^ CPD photolyases were found in the Asian or wild rice species (Fig. S10 and 11), indicating that they are specific to and conserved in African rice species only.

## Discussion

In this study, we investigated the UVB resistance of 15 African rice cultivars with the AA genome, i.e., 7 *O. sativa* (TOS), 2 *O. barthii* (TOB) and 6 *O. glaberrima* (TOG), focusing on CPD photolyase genotypes and activities. Previously, we classified Asian rice cultivars and some wild rice accessions as UVB resistant, sensitive and hypersensitive and found that UVB resistance was correlated with CPD photolyase genotypes^17^. Here, we show that the resistance to UVB radiation also varies widely among African rice cultivars grown under laboratory conditions at a specific UVB intensity (UVB_BE_: 14.7 kJ/m^2^/d) and that they are either UVB-hypersensitive or UVB-super-hypersensitive, i.e., more sensitive than the hypersensitive Asian *O. sativa* cultivar Surjamkhi. This finding was surprising because African rice cultivars are resistant to many biotic and abiotic stresses^15,25,26^. The difference in UVB resistance correlated with the total CPD photolyase activity as determined by the activity of CPD photolyase, which is affected by its genotype, and its content in the cell. The UVB-super-hypersensitive cultivars had relatively low total CPD photolyase activity because of the low activity and content of CPD photolyase. African rice species, even cultivars with the same CPD photolyase genotype, showed large variation in the UVB resistance index. This variation could be caused by different CPD photolyase contents. The CPD photolyase content has been thought to be almost the same among Asian rice cultivars, especially in cultivars of similar genotypes^11,12,17^. However, the CPD photolyase content estimated from *in vitro* CPD photolyase activity and the activity of purified CPD photolyase varied widely not only in African rice cultivars but also in Asian rice cultivars (Fig. S5a). In addition, the differences in CPD photolyase content might have been caused by differences not only in gene transcription but also in protein stability. We previously demonstrated that Arabidopsis CPD photolyase expression is regulated by multiple photoreceptors through cryptochrome-, phytochrome- and UVR8-dependent pathways and by UVB-induced-damage and stress-response signalling pathways as protection against harmful effects of UVB radiation^27^. Therefore, the regulation of CPD photolyase gene expression might differ among domesticated rice cultivars and species adapted to different regions and environments.

We previously purified native rice CPD photolyase 8,100-fold from the leaves of a CPD photolyase-overexpressing transgenic Sasanishiki plant^20^. Native Sasanishiki CPD photolyase has both a pterin-like chromophore and an FAD chromophore. In this study, we purified native CPD photolyase 53,000–165,000-fold from the leaves of each of the three Asian rice cultivars and representative African cultivars with different CPD photolyase genotypes (Table S2). The activity of CPD photolyase (CPD/Mb/min/ng protein) calculated from purified CPD photolyase of the Sasanishiki (UVB-resistant), Norin 1 (UVB-sensitive) or Surjamkhi (UVB-hypersensitive) cultivars was 0.58, 0.50 or 0.26, respectively and was higher than the value for the Sasanishiki CPD photolyase (0.14 CPD/Mb/min/ng protein) we reported previously^20^. This difference between our present and previous studies might be caused by the difference in the samples used in the assay. In a previous study, CPD photolyase was purified from Sasa-type-CPD photolyase-overexpressing transgenic rice plants: the transcript level of CPD photolyase in this overexpressing line was 149-fold higher than that in the wild type, and the increased activity of CPD photolyase in the crude extract was 46-fold^28^. When protein is strongly overexpressed in the cell using transcriptional induction of strong promoters, there is a possibility that abnormal or inactive protein exists in the cells. Therefore, a reduction in the existence of active CPD photolyase in the transgenic plant might cause a decrease in the value of activity per weight of protein. As expected from our previous studies based on *in vitro* activity assays in crude extracts and *in vivo* activity assays in leaves^7^, the activity of native CPD photolyase differs significantly among these Asian cultivars.

In African cultivars belonging to *O. glaberrima* and *O. barthii*, we found novel polymorphisms resulting in the S^78^-R^126^-A^283^-Q^296^ or P^78^-R^126^-A^283^-Q^296^ CPD photolyase variants. The activities and predicted 3D structures of these purified native CPD photolyases were not significantly different from each other, suggesting that the replacement of P^78^ with S did not affect protein activity. By contrast, replacement of G^283^ with A reduced the protein activity with decreasing CPD binding to CPD photolyase, similar to the replacement of Q^296^ with H, as previously reported^21^ (Fig. 4c). The replacement of G with an A residue was expected to generate negligible conformational changes because A is similar in size to the G residue and both are nonpolar amino acids. However, contrary to our expectations, the 3D structure predicted using PyMol showed that this replacement caused structural changes through the loss of polar contacts between rice CPD photolyase and the FAD chromophore. Yang and Li demonstrated that the replacement of a G residue with an A residue at position 168 of a UV-induced spore photoproduct (5-tyminyl-5,6-diydrothymine) repair enzyme, spore photoproduct lyase (SPL), reduces the SPL activity by 3~4-fold relative to that of the WT enzyme^29^. Thus, the replacement of G with A at position 283 of rice CPD photolyase might cause subtle conformational changes that can affect the enzyme activity. In addition, replacement of Q with H at position 296 also affects the binding activity of CPD^21^. The amino acid residue at position 296 is also important for FAD binding^21^, and the substrate (CPD)-binding pocket is located close to the FAD-binding site^22^ (Fig. S7a). Thus, these results indicate that the subtle conformational changes occurring adjacent to the FAD binding site could affect rice CPD photolyase activity resulting from a decrease in CPD binding. A detailed understanding of how structural changes caused by amino acids, such as replacement of G^283^ with A and/or that of P^78^ with S, affect the CPD photolyase structure could possibly be achieved by determining the crystal structure of a CPD-containing duplex African rice CPD photolyase.

Our phylogenetic analysis revealed that the presence of the P^78^-R^126^-A^283^-Q^296^ and S^78^-R^126^-A^283^-Q^296^ CPD photolyases in *O. glaberrima* and *O. barthii* (native African rice) supports the proposition that these genotypes originated from Africa (Fig. 6a). Interestingly, the P^78^-R^126^-A^283^-Q^296^ and S^78^-R^126^-A^283^-Q^296^ genotypes were found only in cultivars from West Africa, largely in countries believed to be centres of domestication of *O. glaberrima* (Mali, Senegambia, Guinea and Guinea Bissau; West African coasts)^30^ (Fig. 6b). The countries of origin of all African *O. sativa* cultivars with genotypes identical to that of the Asian cultivar Surjamkhi (*O. sativa* ssp*. indica*) (P^78^-R^126^-G^283^-H^296^) were distributed widely throughout Africa and included countries proposed as the principal entry zone during the introduction of Asian *O. sativa* into West Africa (Guinea and Guinea Bissau)^31^ (Fig. 6b). Asian *O. sativa* may have been introduced to various areas of Africa, including West Africa, by humans, perhaps during the period of the Atlantic slave trade (beginning ca. 1550) or earlier through trans-Saharan trade routes^32^.

Finally, in this study, to evaluate the UVB resistance of African rice cultivars domesticated in African regions where the UVB intensity is higher than that in the Asian region, we performed experiments under supplemented UVB intensity (UVB_BE_: 14.7 kJ/m^2^/d) under the same conditions as previously performed for classification of UVB sensitivity among Asian rice cultivars. The biologically effective UVB radiation was calculated using the plant action spectrum^33^. This value was higher than the current higher ground level of UVB radiation in a year in the Sonoran Desert, USA (8.0 kJ/m^2^/d)^34^, or in Cape town, South Africa (8.5 kJ/m^2^/d)^35^. Consequently, the African rice cultivars (tropical *O. glaberrima*, *O. barthii*, and *O. sativa*) examined here were UVB-hypersensitive or even UVB-super-hypersensitive in comparison with Asian *O. sativa* cultivars. Although it is not yet certain what kind of influence the nature of UVB-super-hypersensitivity or UVB-hypersensitivity has on the growth or yield of these cultivars domesticated in Africa, it is worth noting that the rice cultivars with UVB-super-hypersensitivity or hypersensitivity caused by lower CPD photolyase activity have been domesticated in Africa, where UVB radiation is high. African rice species (*O. glaberrima*) have two major ecotypes: The floating photosensitive ecotype, grown in deep water, including coastal mangrove areas, and the early erect ecotype, grown in uplands or moderately flooded lowlands^30^. UVB-super-hypersensitive cultivars might be deep-water rice, and most *O. glaberrima* adapt well to floods and long-term submergence due to escape strategies based on the elongation of stems or leaves^36,37^. Therefore, African rice might not need high CPD photolyase activity because UVB radiation does not pass through deep water. However, after the water level begins to decrease or during short-term submergence (flash flooding) due to unstable climatic conditions, adaptation strategies to survive flooding, such as shoot and internode elongation, will make this species vulnerable to not only lodging and photoperiodic sensitivity^36,37^ but also to UVB radiation. This may contribute to the low productivity of *O. glaberrima* because it is grown in a tropical environment with high UVB radiation stress, with unstable climatic conditions that are strongly affected by precipitation and overflow from the river due to the poor irrigation system of rice cultivated in West Africa^37^. Another possibility is that UVB super-hypersensitivity enables cultivation under various environmental stresses or is beneficial for overcoming stresses such as pathogens^38,39^ because a UVB-hypersensitive *indica* cultivar (Surjamkhi) whose CPD photolyase has low activity has been domesticated in South Asia, where UVB radiation is also high. These possibilities should be explored in the future.

In conclusion, our study revealed that the domestication of African rice species may have contributed to the various UVB sensitivity phenotypes, which may enable broad adaptation to multiple environmental stresses. This finding provides key information about the evolutionary history of the CPD photolyase gene in rice, which will offer new tools for the improvement of both Asian and African rice cultivars.

## Methods

### Plant material and growth conditions

The 15 African rice cultivars used in this study were the *O. sativa* cultivars TOS7940 (Accession number WAB0009687), TOS8086 (WAB0009756), TOS8722 (WAB0010137), TOS10589 (WAB0026804), TOS13649 (WAB0025539), TOS13699 (WAB0025692), and TOS14844 (WAB0013700), the *O. barthii* cultivars TOB7307 (WAB0008667) and TOB14466 (WAB0013402), and the *O. glaberrima* cultivars JIAKAWO WODEWO (WAB0004306), MB3 (WAB0009234), TOG12380 (WAB0011910), TOG14928 (WAB0013759), C7251 and MARO GOUDO (WAB0006737). All of them except C7251 were provided by the Rice Biodiversity Center for Africa (http://eservices.africarice.org/argis/index.php; Cotonou, Benin). C7251 was purchased from the Genetic Strain Research Center, National Institute of Genetics (Mishima, Japan). All plants, including 3 control Asian rice cultivars (*O. sativa*) [UVB-resistant Sasanishiki (*japonica*), UVB-sensitive Norin 1 (*japonica*) and UVB-hypersensitive Surjamkhi (*indica*)], were grown and treated with UVB as follows: seeds of each cultivar, soaked in water at 30 °C for 2 d, were sown for 30 d in pots (15 cm wide × 6 cm deep × 10 cm high) containing fertilized soil in a large growth cabinet (Tabai Espec Ltd., Osaka, Japan), with a 12 h/12 h photoperiod and temperatures at 27/17 °C. Five seedlings of each cultivar were grown under visible light supplied by a combination of metal halide lamps (MT 400 DL/BUD; Iwasaki Electric Ltd. Co., Saitama, Japan) and higher-pressure sodium lamps (NH360DL; Iwasaki Electric Ltd. Co.) positioned at the top of the chamber, with a heat-absorbing filter (Tabai Espec Ltd. Co., Osaka, Japan). The heat-absorbing filter eliminated radiation below 350 nm^40^. Photosynthetically active radiation (PAR) was recorded with a data logger (LI-1000; Li-Cor Inc., Lincoln, NE, U.S.A.) and an L1-190SA sensor (Li-Cor Inc.). The PAR was adjusted to approximately 350 µmol photon/m^2^/s at the top of the plants. When necessary, plants were grown under visible light supplemented with UVB radiation using three UVB bulbs (FL20SE; Toshiba, Tokyo, Japan) located above the plants. Plants receiving UVB were subjected to the same photoperiod as that of the plants grown with visible radiation. Under the UVB bulbs, a UV29 glass filter (Toshiba Glass Co., Shizuoka, Japan) reduced 290 nm radiation by 50%^40^. The UVB intensity was measured with a data logger (LI-1000) and an SD-104B sensor (Li-Cor Inc.). The UVB intensity at the plant level was 1.2 W/m^2^. Spectral distribution was measured with a spectroradiometer (USR-45DA: Ushio Inc., Tokyo, Japan). Biologically effective UVB radiation (14.7 kJ/m^2^/d) was calculated using the plant action spectrum of Caldwell^33^ normalized to unity at 300 nm.

To assess growth, plants were grown for 21 days under visible radiation with or without supplementary UVB radiation in a growth chamber, the tillers were counted, and the above-ground parts were weighed. The value of the middle three of five plants was used for calculating the following scores because poorly performing plants appeared occasionally regardless of UVB treatment. The UVB resistance index was calculated by multiplying the sum of the values of the ratio of irradiated to unirradiated tiller number and the above-ground fresh weight by 100^18^. To measure the level of transcription of the CPD photolyase gene, photorepair activity using crude extract and activity of CPD photolyase, seedlings were grown for 16 days under visible radiation in a large growth cabinet until the third leaves had expanded fully, and then the third leaves were harvested and used in the experiments. Whole plants were used for purification of CPD photolyase protein and measurements of activity.

### Purification of CPD photolyase from rice plants

The CPD photolyase purification methods were modified from Teranishi *et al*.^20^. All steps were carried out at 0 °C to 4 °C under dim red light. Protein concentration was determined according to the method published by Bradford^41^ or estimated from the intensity of the bands on SDS polyacrylamide gels (SDS-PAGE) stained with SYPRO Ruby protein gel stain (Bio-Rad). Band intensity was quantified with Image Lab 6.0 software (Bio-Rad). In both methods, bovine serum albumin (BSA) (Seikagaku Co., Tokyo, Japan) was used as a standard.

Whole plants (20 g) were homogenized in 100 mL buffer A [160 mM potassium phosphate (pH 7.2), 5 mM EDTA, 2 mM dithiothreitol (DTT) and 10% (v/v) glycerol] in a chilled mortar with a pestle. The homogenate was centrifuged at 27,000 × *g* for 30 min at 4 °C, and the supernatant was used as a crude extract (fraction 1).

Ammonium sulphate (20.8 g) was added to 100 mL of fraction 1 with magnetic stirring to a final concentration of approximately 35% saturation. The mixture was centrifuged as above, and the precipitate was discarded. Ammonium sulphate (33.0 g) was added to 140 mL of the supernatant with gentle magnetic stirring to a final ammonium sulphate concentration of approximately 70% saturation, and the mixture was centrifuged as above. The protein precipitate was dissolved in 30 mL buffer A and dialyzed overnight against buffer B [40 mM potassium phosphate (pH 7.2), 5 mM EDTA, 2 mM DTT, 10% (v/v) glycerol, and 80 mM NaCl] to produce fraction 2.

Fraction 2 (35 mL) was loaded onto an UNO-Q12 anion-exchange column (1.5 cm × 6.8 cm; Bio-Rad) and eluted with buffer B at a flow rate of 2 mL/min. CPD photolyase was recovered in the flow-through (fraction 3). Proteins bound to the UNO-Q12 column were eluted with buffer C [40 mM potassium phosphate (pH 7.2), 5 mM EDTA, 2 mM DTT, 10% (v/v) glycerol, 1 M NaCl] at a flow rate of 2 mL/min.

Fraction 3 (60 mL) was loaded onto a heparin affinity column (1.6 cm × 10 cm; GE Healthcare UK), and the column was washed with buffer B at a flow rate of 1 mL/min. Protein bound to the column was eluted with buffer C at the same flow rate and was dialyzed overnight against buffer B. CPD photolyase was recovered in this fraction (fraction 4).

Finally, CPD photolyase was purified with UV-irradiated DNA-conjugated magnetic beads. The surfaces of all tubes and magnetic beads used in the final purification were blocked by incubating them 2 or 3 times with 0.1 mg/mL BSA, followed by 2 or 3 washes with buffer B to remove unbound BSA. A 5′-biotinylated 42-mer oligonucleotide (5′-ATGGCGCCAGACGTACTAATGTGTATACACGCGTGCATGATC-3′) and a complementary unmodified 42-mer oligonucleotide (5′-GATCATGCACGCGTGTATACACATTAGTACGTCTGGCGCCAT-3′) were synthesized by Kurabo Industries. To generate CPDs, the unmodified oligonucleotide [100 nmol/mL in 1 × Tris-EDTA (TE) buffer (10 mM Tris-HCl, pH 8.0, 1 mM EDTA)] was irradiated with a germicidal lamp (Toshiba; 254 nm, 10 W/m^2^) for 8 h and was then mixed with the same amount of the 5′-biotinylated oligonucleotide. The mixture was boiled for 5 min and allowed to anneal by cooling slowly to room temperature. The resulting double-stranded DNA (1.5 nmol) was conjugated to 6 mg streptavidin-coated magnetic beads (Magnotex-SA; Takara Bio Inc., Shiga, Japan). Fraction 4 (5 mL) was mixed with 0.3 mL UV-irradiated DNA conjugated to magnetic beads and incubated at 4 °C for ≥8 h. The beads were collected with a magnet, washed five times with 1 mL buffer B, and bound proteins were eluted with buffer C to recover CPD photolyase (fraction 5). The eluate was dialyzed overnight against buffer D [40 mM potassium phosphate (pH 7.2), 5 mM EDTA, 2 mM DTT, 10% (v/v) glycerol, and 4 mM NaCl]. The dialysate was concentrated 10 times under N_2_ flow on ice in the dark and was dialyzed overnight against buffer B. Proteins were separated by SDS-PAGE in 7.5% or 12.5% (w/v) gels and stained with SYPRO Ruby. The band intensity was quantified with Image Lab 6.0.

### DNA sequencing

The procedure for DNA extraction has been previously described^17^. Each PCR mixture contained genomic DNA (1 µg) as a template, 0.2 mM dNTPs, 2.0 mM MgCl_2_, 0.02 U/mL Ex *Taq* polymerase (Takara Bio Inc.) and 100 nM each of the following sets of primers. The primers Glf-F1 (5′-CACAAACGCACGCCCGCA-3′) and GSP4 (5′-GGCTCACACCAGTCAATCTCCGGC-3′) were used to amplify a 1147-bp DNA fragment (exons 1–4 of the rice CPD photolyase gene). The primers H1 (5′-GCGTCGGCGAAGATGGAGTAT-3′) and Rice3 (5′-CCGAGCTCGTGGTATACCACACAAAGAAATG-3′) were used to amplify a 2878-bp DNA fragment (exons 3–10). PCR was performed in a thermal cycler (Bio-Rad) for 35 cycles of 98 °C for 20 s, 70 °C for 30 s and 72 °C for 2 min, followed by a final extension at 72 °C for 10 min. The PCR products were treated with ExoSAP-IT (Amersham Biosciences) to remove primers and free dNTPs and sequenced using the BigDye Terminator Cycle Sequencing Kit (Applied Biosystems, Norwalk, CT, USA).

### Measurement of CPD photolyase activity

Fully expanded third leaves (0.08 g) were homogenized in 400 µl 40 mM potassium phosphate, pH 7.2, 5 mM EDTA, 2 mM DTT, 0.2 g/L BSA, and 10% (v/v) glycerol using a chilled mortar and pestle. The homogenate was centrifuged for 20 min at 20,000 × *g* and 4 °C, and the supernatant was desalted by passage through a Bio-Gel P6DG spin-column (Bio-Rad) and used as a crude extract to measure photolyase activity. The total soluble protein content was determined according to the method published by Bradford^41^ using BSA as the standard.

*In vitro* photolyase activity was measured as described in detail in ref. ^42^. CPD frequencies were determined using a DNA damage analysis system (Tohoku Electric Co., Miyagi, Japan) as described previously^43^. CPD frequencies (CPD/Mb) were calculated using a molecular length standard curve, and the quantity of DNA at each migration position was determined by quantitative imaging^44,45^.

### Transcript levels of CPD photolyase enzyme; RNA extraction, cDNA synthesis and quantitative real-time RT PCR analysis

Total RNA was extracted from whole seedlings with an RNeasy Plant Mini Kit from Qiagen and treated with DNase I (Qiagen). Reverse transcription was performed with an oligo(dT) primer and a random 6-mer mixture using a Prime Script RT Reagent Kit (Takara Bio Inc.). The PCR primers were 5′-CCGTCGATGCTTTCTTGGAGG-3′ and 5′-CATCTCCAACTGCGATGCATTCCA-3′, which amplify the coding region of CPD photolyase (nucleotides 935 to 1159). The actin gene was used as an internal control, and the actin primers were 5′-GAAGATCACTGCCTTGCTCC-3′ and 5′-CGATAACAGCTCCTCTTGGC-3′. Real-time PCR was performed using SYBR Green to monitor double-stranded DNA synthesis (CFX96, Bio-Rad). The amounts of cDNA were calculated by the delta CT method.

### Purification of the *E. coli*-expressed rice CPD photolyase

The plasmid pGEXOsPHR was constructed as previously described^46^. This plasmid encodes an N-terminal fusion of glutathione *S-*transferase containing a thrombin protease recognition site to CPD photolyase derived from a cDNA sequence from the rice cultivar ‘Sasanishiki’. An inverse, PCR-based, site-directed mutagenesis reaction was performed using a KOD-Plus-Mutagenesis Kit (Toyobo Co., Ltd.) according to the manufacturer’s instructions. The *E. coli* strain KY20 (JM107 + *phr*2*0*∷Kan) was transformed with pGEXOsPHR (Sasanishiki), pGEXOsPHR (P^78^-R^126^-A^283^-Q^296^) and pGEXOsPHR (S^78^-R^126^-A^283^-Q^296^) and grown as previously described^46^. All subsequent steps were carried out at 0 °C to 4 °C under dim red light. Cells were harvested and resuspended in buffer B and disrupted by sonication for 30 min. Sonicated cells were centrifuged at 27,000 × *g* for 30 min at 4 °C, and the supernatant was collected and loaded onto a Glutathione-Sepharose 4B column (1 cm × 5 cm, containing 5 mL resin; GE Healthcare UK). The column was washed with buffer B. The outlet of the column was then closed, and buffer B containing 10 units/mL thrombin protease was added. The column was closed at the top and incubated at 25 °C for 12 h. After protease digestion, the solution, including CPD photolyase, was eluted from the column.

### Measurement of absorption

Absorption spectra of the purified native rice CPD photolyase and the *E. coli-*expressed rice CPD photolyase were obtained with a spectrophotometer (JASCO V-550).

### Isotopic labelling of DNA and electrophoretic mobility shift assay

A 30-mer oligonucleotide (5′-CACGTACGCATCTTCTACGTACCGACAGTC-3′) containing or non-containing a centrally located thymine dimer was used in this experiment^47^. Sixty picomoles of oligonucleotide containing or not containing a thymine dimer were reacted with [*γ*-^32^P]ATP using T4 polynucleotide kinase (TaKaRa Bio Inc.) for 30 min at 37 °C. Unincorporated [*γ*-^32^P]ATP was removed using a Sephadex G-25 column (GE Healthcare UK). The oligonucleotide was then annealed with the complementary oligonucleotide (5′-GACTGTCGGTACGTAGAAGATGCGTACGTG-3′) in annealing buffer (10 mm Tris-HCl [pH 8.3] and 10 mm MgCl_2_) by heating at 95 °C for 5 min and cooling to 30 °C over a 30-min period. The duplex DNA was precipitated with ethanol and resuspended in 1 × TE buffer. Zero to 400 ng of the *E. coli-*expressed rice CPD photolyase was added to 0.3 pmol of the ^32^P-labelled DNA substrates in the binding buffer (40 mm potassium phosphate buffer, pH 7.2, 5 mm EDTA, 2 mm DTT, and 80 mm NaCl) in the dark at 25 °C for 15 min. Then, electrophoresis was performed in a 10% (w/v) nondenatured polyacrylamide gel. After electrophoresis, the band intensity of the radiolabelled oligonucleotides on the gel was measured with a fluoro-image analyser (FLA-2000; Fuji Photo Film).

### Visualization of the CPD photolyase 3D structure by PyMol software

PyMol 2.2.2 software from Schrodinger was used as a comprehensive software package for rendering and animating 3D structures by using amino sequence information of the selected cultivar TOB7307. Then, the CPD 3D structure was constructed focused on amino acid interest in cultivar TOB7307 compared to that of Sasanishiki (protein data bank code: 3UMV)^22^.

### Phylogenetic analysis

Genomic sequences of the Asian rice cultivars Sasanishiki, Norin 1 and Surjamkhi were obtained from the DDBJ/EMBL/GenBank database (accession nos. AB096003, AB099694 and AB125340, respectively). The orthologous sequences of other rice species were obtained from the EMBL-EBI database under the following transcript IDs: Os10t0167600-02 for *Oryza sativa* ssp. *japonica*, OB10G12750 for *Oryza brachyantha*, ORGLA10G0028500.1 for *Oryza glaberrima*, OBART10G03590 for *Oryza barthii*, OGLUM10G03610.1 for *Oryza glumaepatula*, KN540024.1_FGT005 for *Oryza longistaminata*, OMERI10G02990.1 for *Oryza meridionalis*, ONIVA12G06340.1 for *Oryza nivara*, OPUNC10G03030.1 for *Oryza punctata*, ORUFI10G03840.1 for *Oryza rufipogon*, BGIOSGA032246 for *Oryza sativa* ssp*. indica* and LPERR10G02700.1 for *Leersia perrieri*. In total, 29 nucleotide sequences (761 positions per sequence after elimination of positions containing gaps and missing data) were included in the analysis. The evolutionary history was inferred using the UPGMA method^48^ in MEGA7^49^. The optimal tree had a sum of branch lengths of 159.1. The tree was drawn to scale, with branch lengths in the same units as those of the evolutionary distances used to infer the tree, which were computed using the number of differences method^50^ and are in the units of the number of base differences per sequence.

### Statistical analyses

Statistical analyses were performed using Microsoft Office Excel 2016 (Microsoft Co., Redmond, WA, USA), GraphPad Prism version 8.00 (GraphPad Software, San Diego, CA, USA) and R programming language (https://www.r-project.org/).

## Supplementary information

Supplementary Figures and Tables.

## References

[1] Teramura AH (1983). Effects of ultraviolet‐B radiation on the growth and yield of crop plants. Physiol. Plant..

[2] Krizek DT, Mirecki RM, Britz SJ (1997). Inhibitory effects of ambient levels of solar UV-A and UV-B radiation on growth of cucumber. Physiol. Plant..

[3] Krizek DT, Britz SJ, Mirecki RM (1998). Inhibitory effects of ambient levels of solar UV-A and UV-B radiation on growth of cv. New Red Fire lettuce. Physiologia Plantarum.

[4] Meyer RS (2016). Domestication history and geographical adaptation inferred from a SNP map of African rice. Nat. Genet..

[5] Wang M (2014). The genome sequence of African rice (*Oryza glaberrima*) and evidence for independent domestication. Nat. Genet..

[6] Sato T, Kumagai T (1993). Cultivar differences in resistance to the inhibitory effects of near-UV radiation among Asian ecotype and Japanese lowland and upland cultivars of rice (*Oryza sativa* L.). Japanese J. Breed..

[7] Hidema J, Kumagai T (2006). Sensitivity of rice to ultraviolet-B radiation. Ann. Bot.

[8] Sancar A (1994). Mechanisms of DNA excision repair. Science.

[9] Britt AB (1996). DNA damage and repair in plants. Annu. Rev. Plant Biol..

[10] Hidema J, Kumagai T, Sutherland JC, Sutherland BM (1997). Ultraviolet B-sensitive rice cultivar deficient in cyclobutyl pyrimidine dimer repair. Plant Physiol..

[11] Hidema J, Kumagai T, Sutherland BM (2000). UV radiation-sensitive norin 1 rice contains defective cyclobutane pyrimidine dimer photolyase. Plant Cell.

[12] Hidema J (2005). Spontaneously occurring mutations in the cyclobutane pyrimidine dimer photolyase gene cause different sensitivities to ultraviolet-B in rice. Plant J.

[13] Jusu, M. S. Management of genetic variability in rice (*Oryza sativa* L. and *O. glaberrima* Steud.) by breeders and farmers in Sierra Leone. PhD Thesis, Technology & Agrarian Development Group (Wageningen University & Research Centre, 1999).

[14] Brar, D. S. & Khush, G. S. Alien introgression in rice. In Oryza: from molecule to plant 35–47 (Springer, 1997). 9291958

[15] Sanchez, P. L., Wing, R. A. & Brar, D. S. The wild relative of rice: genomes and genomics. in *Genetics and genomics of rice* 9–25 (Springer, 2013).

[16] Second, G. A New insight into the genome differentiation in *Oryza* L. through isozymic studies. A comparison with similar studies in Dactylis and Triticum. (eds. Sharma, A.K. & Sharma, A.) 45–78 (*Adv. Chromosom. Cell Genet.* University Press, New York, 1985).

[17] Iwamatsu Y (2008). UVB sensitivity and cyclobutane pyrimidine dimer (CPD) photolyase genotypes in cultivated and wild rice species. Photochem. Photobiol. Sci..

[18] Barnes PW, Maggard S, Holman SR, Vergara BS (1993). Intraspecific variation in sensitivity to UV-B radiation in rice. Crop Sci..

[19] Teranishi M, Iwamatsu Y, Hidema J, Kumagai T (2004). Ultraviolet-B sensitivities in Japanese lowland rice cultivars: cyclobutane pyrimidine dimer photolyase activity and gene mutation. Plant Cell Physiol.

[20] Teranishi M, Nakamura K, Morioka H, Yamamoto K, Hidema J (2008). The native cyclobutane pyrimidine dimer photolyase of rice is phosphorylated. Plant Physiol..

[21] Yamamoto A (2007). Biochemical and biological properties of DNA photolyases derived from utraviolet-sensitive rice cultivars. Genes Genet. Syst..

[22] Hitomi K (2012). Eukaryotic class II cyclobutane pyrimidine dimer photolyase structure reveals basis for improved ultraviolet tolerance in plants. J. Biol. Chem..

[23] Zou XH (2008). Analysis of 142 genes resolves the rapid diversification of the rice genus. Genome Biol..

[24] Ammiraju JSS (2005). The Oryza bacterial artificial chromosome library resource: Construction and analysis of 12 deep-coverage large-insert BAC libraries that represent the 10 genome types of the genus Oryza. Genome Res..

[25] Second G (1985). Evolutionary relationships in the *sativa* group of *Oryza* based on isozyme data. Genet Sel Evol.

[26] Takeoka T (1965). Taxonomy and chromosome numbers of African representatives of the *Oryza officinalis* complex. Shokubutsugaku Zasshi.

[27] Li N (2015). UV-B-induced CPD photolyase gene expression is regulated by UVR8-dependent and-independent pathways in arabidopsis. Plant Cell Physiol.

[28] Hidema J (2007). Increase in CPD photolyase activity functions effectively to prevent growth inhibition caused by UVB radiation. Plant J.

[29] Yang L, Li L (2017). Insights into the activity change of spore photoproduct lyase induced by mutations at a peripheral glycine residue. Front. Chem.

[30] Semon M, Nielsen R, Jones MP, McCouch SR (2005). The population structure of African cultivated rice *Oryza glaberrima* (Steud.): evidence for elevated levels of linkage disequilibrium caused by admixture with *O. sativa* and ecological adaptation. Genetics.

[31] Portères, R. Primary cradles of agriculture in the African content: Papers in African prehisttory (eds. Fage, J.D. & Olivier, R.A.) 43–58 (Cambridge University Press, Cambridge, 1970).

[32] Mokuwa A (2013). Robustness and strategies of adaptation among farmer varieties of African rice (*Oryza glaberrima*) and Asian rice (*Oryza sativa*) across West Africa. PLoS One.

[33] Caldwell MM, Solar U (1971). V. irradiation and the growth and development of higher plants. Photophysiology.

[34] Bornman JF (2019). Linkages between stratospheric ozone, UV radiation and climate change and their implications for terrestrial ecosystems. Photochem. Photobiol. Sci..

[35] Chimphango SBM, Musil CF, Dakora FD (2004). Impact of increased ultraviolet-B radiation due to stratospheric ozone depletion on N_2_ fixation in traditional African commercial legumes. South African J. Bot..

[36] Sakagami J-I, Kawano N (2012). Survival of submerged rice in a flood-prone region of West. Africa. Tropics.

[37] Sakagami, J.-I. Submergence Tolerance of Rice Species, *Oryza glaberrima* Steudel, Applied Photosynthesis, (ed. Mohammad, M. Najafpour) 353–364 (IntechOpen, Available from, https://www.intechopen.com/books/applied-photosynthesis/submergence-tolerance-of-rice-species-oryza-glaberrima-steud-, 2012)

[38] Kunz BA (2008). UV-induced DNA damage promotes resistance to the biotrophic pathogen hyaloperonospora parasitica in *Arabidopsis*. Plant Physiol..

[39] Qi J (2018). Ultraviolet-B enhances the resistance of multiple plant species to lepidopteran insect herbivory through the jasmonic acid pathway. Sci. Rep.

[40] Kang HS, Hidema J, Kumagai T (1998). Effects of light environment during culture on UV-induced cyclobutyl pyrimidine dimers and their photorepair in rice (*Oryza sativa* L.). Photochem. Photobiol..

[41] Bradford MM (1976). A rapid and sensitive method for the for the quantitation of microgram quantities of protein utilizing the principle of protein dye-binding. Anal. Biochem..

[42] Hada H, Hidema J, Maekawa M, Kumagai T (2003). Higher amounts of anthocyanins and UV-absorbing compounds effectively lowered CPD photorepair in purple rice (O*ryza sativa* L.). Plant, Cell Environ.

[43] Hidema J, Kumagai T (1998). UVB-induced cyclobutyl pyrimidine dimer and photorepair with progress of growth and leaf age in rice. J. Photochem. Photobiol. B Biol.

[44] Quaite FE, Sutherland BM, Sutherland JC (1992). Quantitation of pyrimidine dimers in DNA from UVB-irradiated alfalfa (*Medicago sativa* L.) seedlings. Appl Theor Electrophor.

[45] Quaite FE, Sutherland JC, Sutherland BM (1994). Isolation of high-molecular-weight plant DNA for DNA damage quantitation: relative effects of solar 297 nm UVB and 365 nm radiation. Plant Mol. Biol..

[46] Hirouchi T (2003). A gene for a class II DNA photolyase from *Oryza sativa*: Cloning of the cDNA by dilution-amplification. Mol. Genet. Genomics.

[47] Iwai S (1994). Endonuclease V from bacteriophage T4 interacts with its substrate in the minor groove. Biochemistry.

[48] Robert, R. Sokal & Sneath Peter, H. A. Numerical taxonomy: the principles and practice of numerical classification (W.H. Freeman & Co, 1973).

[49] Kumar S, Stecher G, Tamura K (2016). MEGA7: Molecular evolutionary genetics analysis Version 7.0 for bigger datasets. Mol. Biol. Evol..

[50] Nei, M. & Kumar, S. *Molecular evolution and phylogenetics*. (Oxford University Press, Oxford, 2000).

