# Supplementary material for: Very high sensitivity of African rice to artificial ultraviolet-B radiation caused by genotype and quantity of cyclobutane pyrimidine dimer photolyase
Source: Sci Rep. 2020 Feb 21;10:3158. doi: 10.1038/s41598-020-59720-x (PMC7035317; doi:10.1038/s41598-020-59720-x)
Supplement: Supplementary file 1 — Supplementary Figures and Tables. [file 41598_2020_59720_MOESM1_ESM.pdf]

SUPPLEMENTARY INFORMATION

Manuscript tittle: Very high sensitivity of African rice to artificial ultraviolet-B radiation caused by genotype and quantity of cyclobutane pyrimidine dimer photolyase

Authors: Gideon S. Mmbando, Mika Teranishi, Jun Hidema*.

Corresponding author: Jun Hidema
Department of Molecular and Chemical Life Sciences, Graduate School of Life Sciences, Tohoku University, Sendai 980-8577, Japan
Telephone: +81 22 217 5690, Fax: +81 22 217 5691, E-mail: j-hidema@ige.tohoku.ac.jp

Supplementary Figure S1

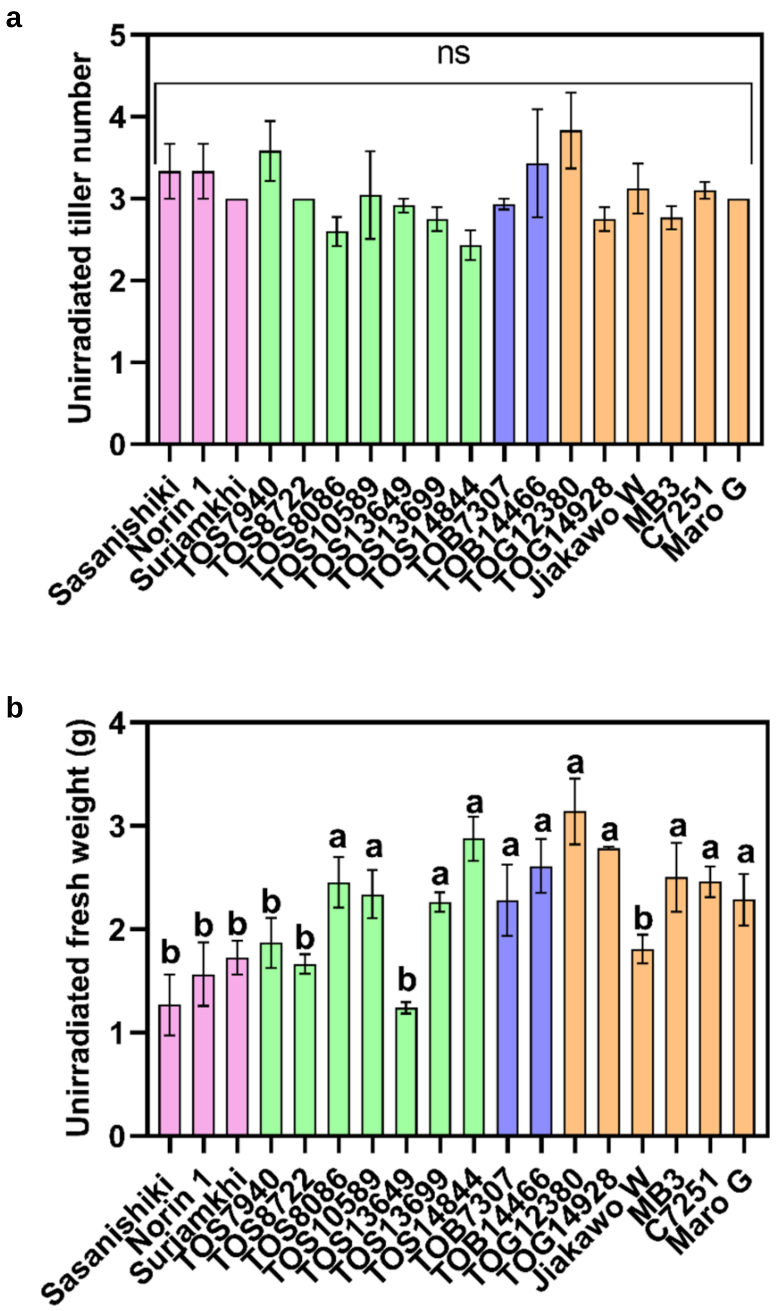

Figure S1. Tiller number and fresh weight of unirradiated UVB radiation plants. Plants of three rice species (*Oryza sativa*, *O. barthii* and *O. glaberrima*) were grown in a growth cabinet for 21 days without (-UVB) UVB radiation. Jiakawo W, Jiakawo Wodewo; Maro G, Maro Goudo. **a** , Tiller number of plant grown under –UVB condition. **b**, Fresh weight of plant grown under –UVB. Unirradiated fresh weight varied widely among African rice cultivars. Values are means ± SD. n=9 repeats; different letters indicate significant differences determined by the Tukey-Kramer test ($P < 0.05$); ns, not significant. Colour coding is as in Fig. 1.

Supplementary Figure S2

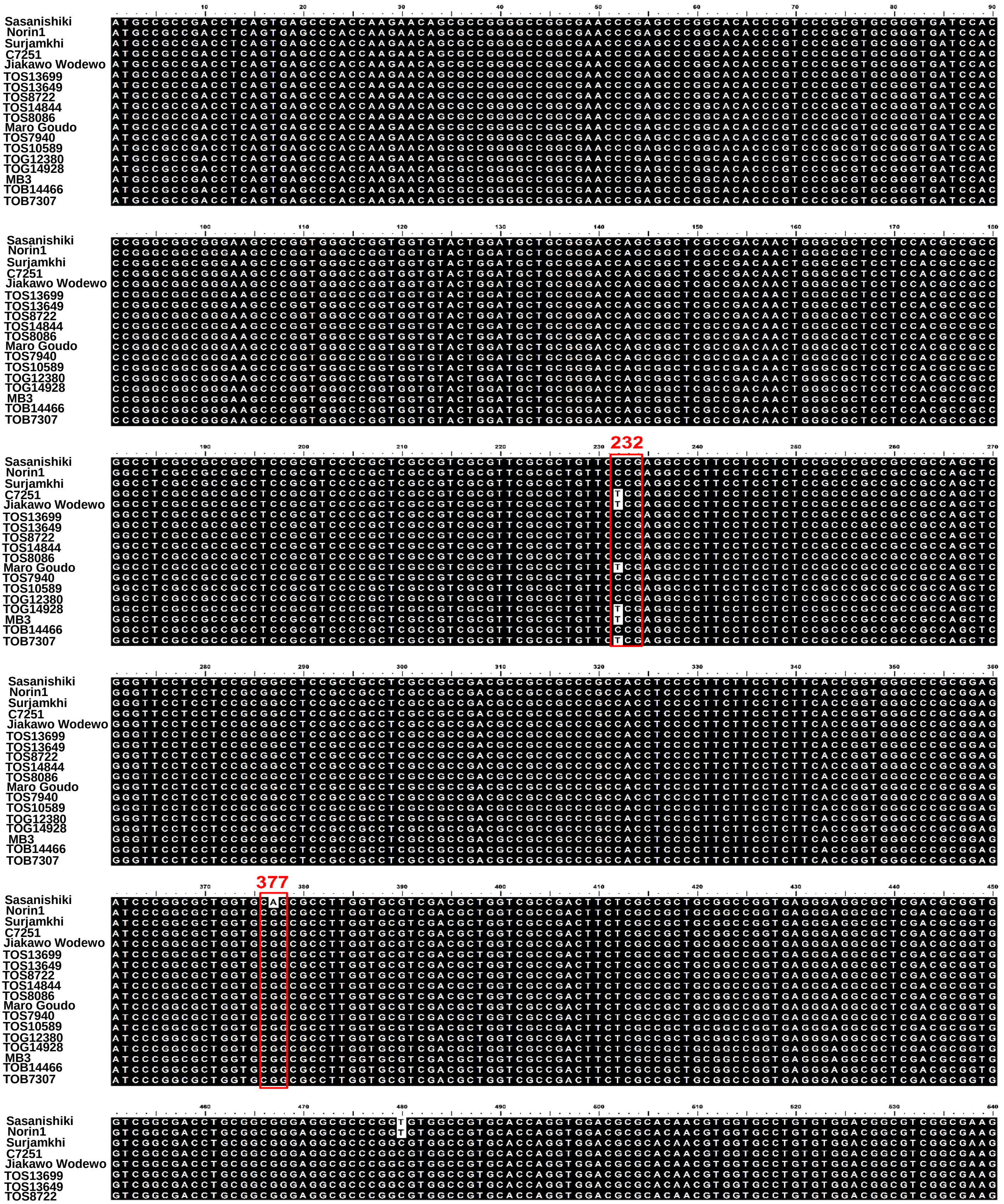

Supplementary Figure S2 **continued**

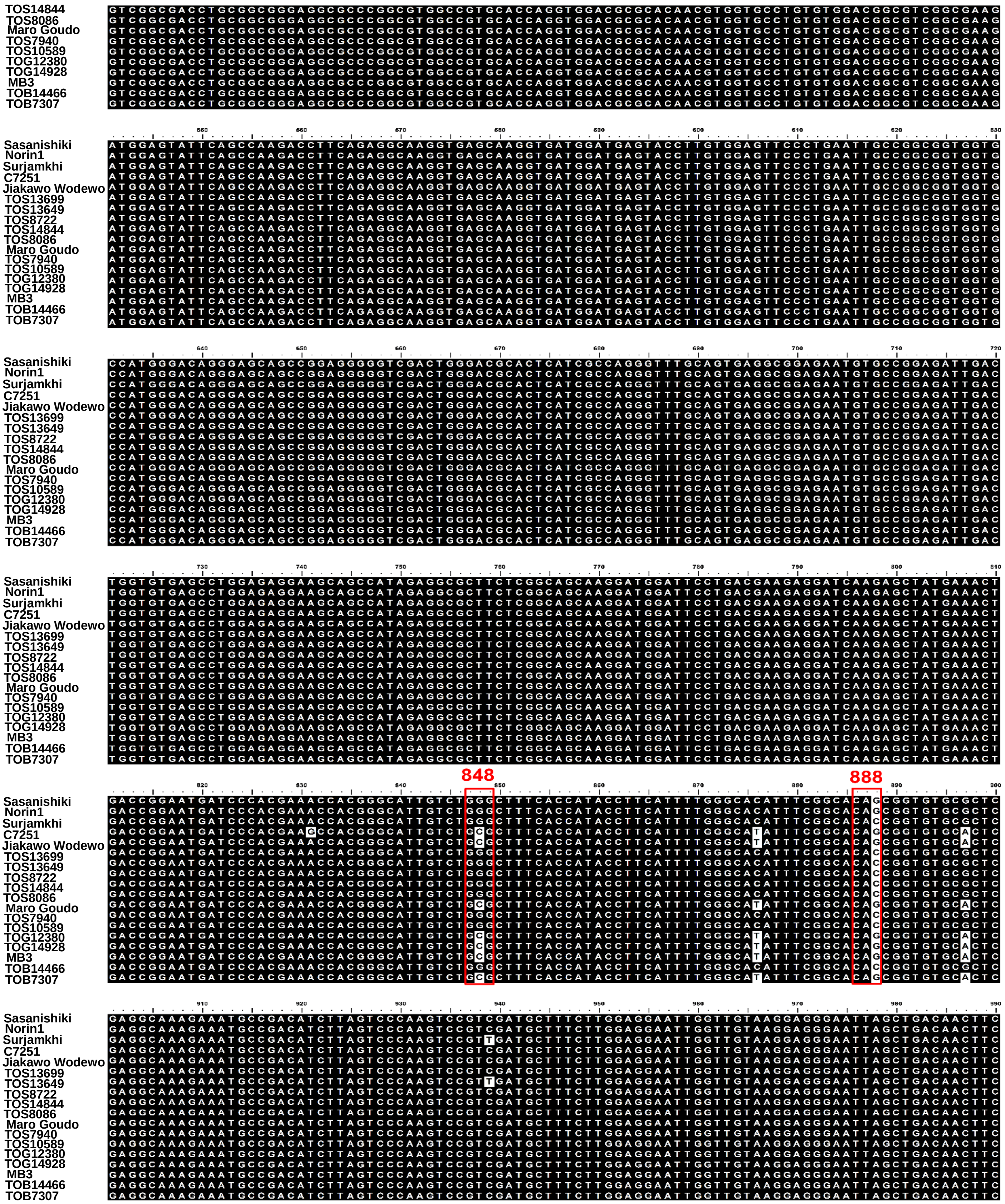

Supplementary Figure S2 **continued**

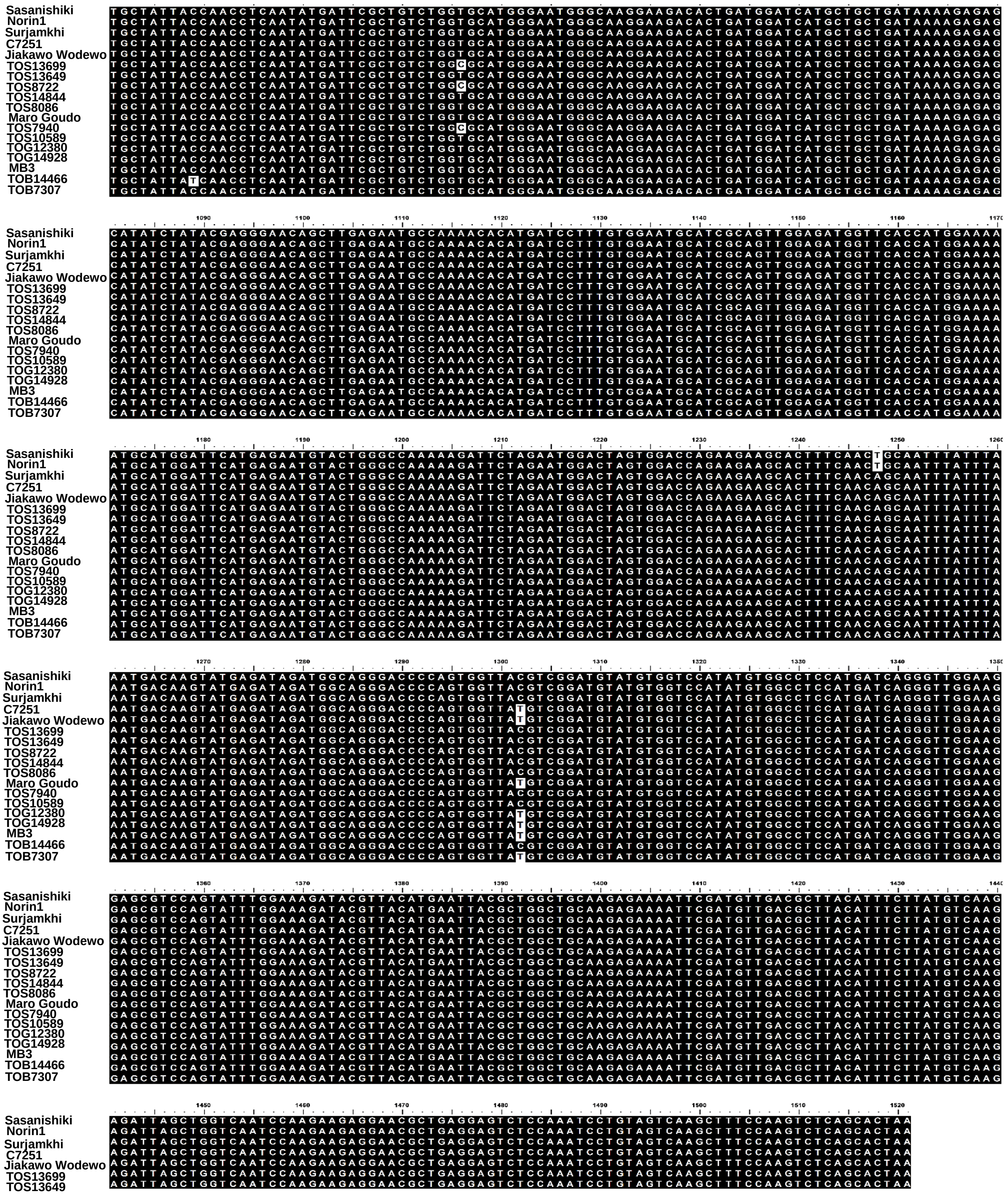

Supplementary Figure S2 **continued**

TOS8722	AGATTAGCTGGTCAATCCAAGAAGAGGAACGCTGAGGAGTCTCCAAATCCTGTAGTCAAGCTTTCCAAGTCTCAGCACTAA
TOS14844	AGATTAGCTGGTCAATCCAAGAAGAGGAACGCTGAGGAGTCTCCAAATCCTGTAGTCAAGCTTTCCAAGTCTCAGCACTAA
TOS8086	AGATTAGCTGGTCAATCCAAGAAGAGGAACGCTGAGGAGTCTCCAAATCCTGTAGTCAAGCTTTCCAAGTCTCAGCACTAA
Maro Goudo	AGATTAGCTGGTCAATCCAAGAAGAGGAACGCTGAGGAGTCTCCAAATCCTGTAGTCAAGCTTTCCAAGTCTCAGCACTAA
TOS7940	AGATTAGCTGGTCAATCCAAGAAGAGGAACGCTGAGGAGTCTCCAAATCCTGTAGTCAAGCTTTCCAAGTCTCAGCACTAA
TOS10589	AGATTAGCTGGTCAATCCAAGAAGAGGAACGCTGAGGAGTCTCCAAATCCTGTAGTCAAGCTTTCCAAGTCTCAGCACTAA
TOG12380	AGATTAGCTGGTCAATCCAAGAAGAGGAACGCTGAGGAGTCTCCAAATCCTGTAGTCAAGCTTTCCAAGTCTCAGCACTAA
TOG14928	AGATTAGCTGGTCAATCCAAGAAGAGGAACGCTGAGGAGTCTCCAAATCCTGTAGTCAAGCTTTCCAAGTCTCAGCACTAA
MB3	AGATTAGCTGGTCAATCCAAGAAGAGGAACGCTGAGGAGTCTCCAAATCCTGTAGTCAAGCTTTCCAAGTCTCAGCACTAA
TOB14466	AGATTAGCTGGTCAATCCAAGAAGAGGAACGCTGAGGAGTCTCCAAATCCTGTAGTCAAGCTTTCCAAGTCTCAGCACTAA
TOB7307	AGATTAGCTGGTCAATCCAAGAAGAGGAACGCTGAGGAGTCTCCAAATCCTGTAGTCAAGCTTTCCAAGTCTCAGCACTAA

Figure S2. Genomic sequences of the CPD photolyase genes of African rice species examined in this study in comparison with *O. sativa* Sasanishiki, Norin 1 and Surjamkhi. Red boxes indicate nucleotide changes leading to amino acid changes.

Supplementary Figure S3

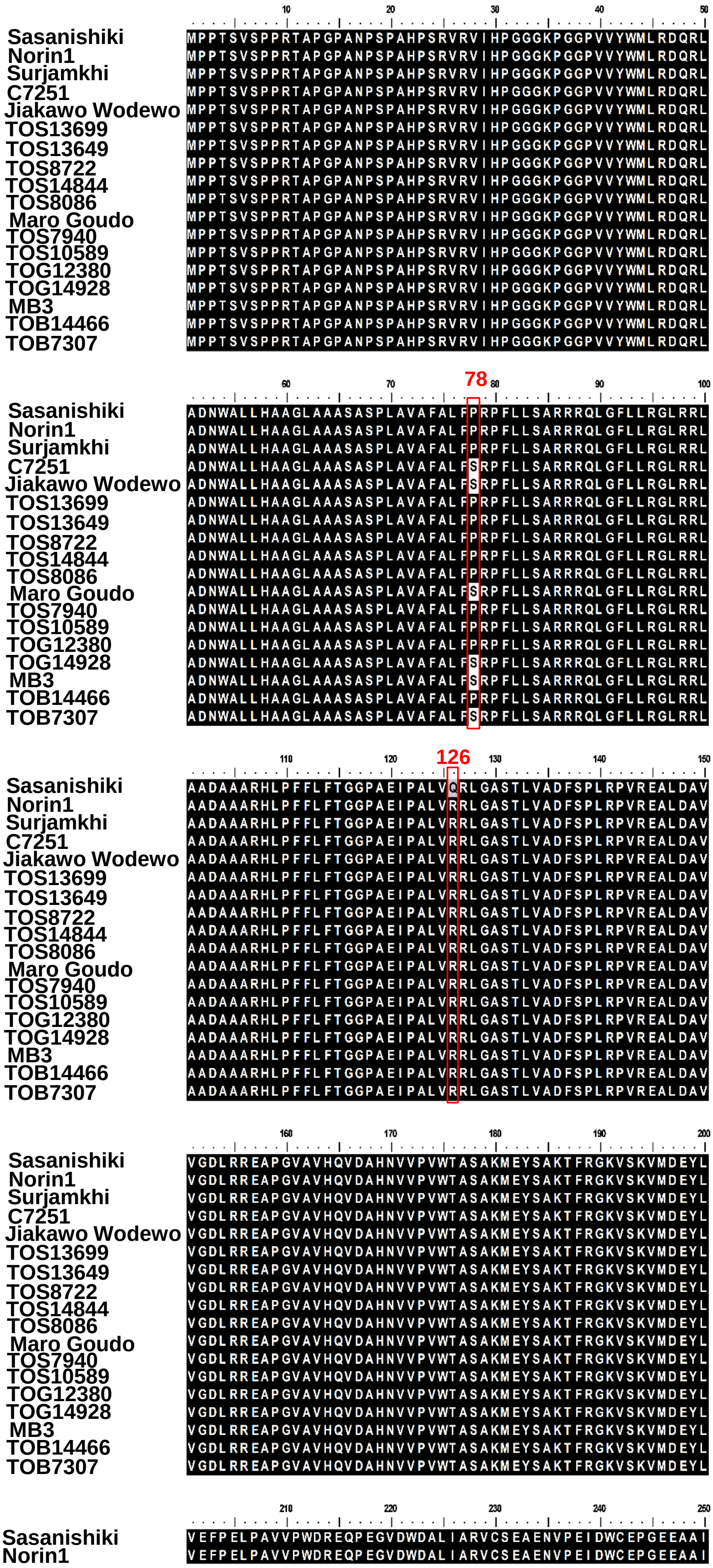

Supplementary Figure S3 continued

Surjamkhi	VEFPELPAVVPWDREQPEGVDWDALIARVCSEAENVPEIDWCEPGEEAAI
C7251	VEFPELPAVVPWDREQPEGVDWDALIARVCSEAENVPEIDWCEPGEEAAI
Jiakawo Wodewo	VEFPELPAVVPWDREQPEGVDWDALIARVCSEAENVPEIDWCEPGEEAAI
TOS13699	VEFPELPAVVPWDREQPEGVDWDALIARVCSEAENVPEIDWCEPGEEAAI
TOS13649	VEFPELPAVVPWDREQPEGVDWDALIARVCSEAENVPEIDWCEPGEEAAI
TOS8722	VEFPELPAVVPWDREQPEGVDWDALIARVCSEAENVPEIDWCEPGEEAAI
TOS14844	VEFPELPAVVPWDREQPEGVDWDALIARVCSEAENVPEIDWCEPGEEAAI
TOS8086	VEFPELPAVVPWDREQPEGVDWDALIARVCSEAENVPEIDWCEPGEEAAI
Maro Goudo	VEFPELPAVVPWDREQPEGVDWDALIARVCSEAENVPEIDWCEPGEEAAI
TOS7940	VEFPELPAVVPWDREQPEGVDWDALIARVCSEAENVPEIDWCEPGEEAAI
TOS10589	VEFPELPAVVPWDREQPEGVDWDALIARVCSEAENVPEIDWCEPGEEAAI
TOG12380	VEFPELPAVVPWDREQPEGVDWDALIARVCSEAENVPEIDWCEPGEEAAI
TOG14928	VEFPELPAVVPWDREQPEGVDWDALIARVCSEAENVPEIDWCEPGEEAAI
MB3	VEFPELPAVVPWDREQPEGVDWDALIARVCSEAENVPEIDWCEPGEEAAI
TOB14466	VEFPELPAVVPWDREQPEGVDWDALIARVCSEAENVPEIDWCEPGEEAAI
TOB7307	VEFPELPAVVPWDREQPEGVDWDALIARVCSEAENVPEIDWCEPGEEAAI

260 270 280 **283** 290 **296** 300

Sasanishiki	EALLGSKDGFLTKRIKSYETDRNDPTKPRALSGLSPYLHFGHISAQRCAL
Norin1	EALLGSKDGFLTKRIKSYETDRNDPTKPRALSGLSPYLHFGHISAQRCAL
Surjamkhi	EALLGSKDGFLTKRIKSYETDRNDPTKPRALSGLSPYLHFGHISAHRCAL
C7251	EALLGSKDGFLTKRIKSYETDRNDPTKPRALSALSPYLHFGHISAQRCAL
Jiakawo Wodewo	EALLGSKDGFLTKRIKSYETDRNDPTKPRALSALSPYLHFGHISAQRCAL
TOS13699	EALLGSKDGFLTKRIKSYETDRNDPTKPRALSGLSPYLHFGHISAHRCAL
TOS13649	EALLGSKDGFLTKRIKSYETDRNDPTKPRALSGLSPYLHFGHISAHRCAL
TOS8722	EALLGSKDGFLTKRIKSYETDRNDPTKPRALSGLSPYLHFGHISAHRCAL
TOS14844	EALLGSKDGFLTKRIKSYETDRNDPTKPRALSGLSPYLHFGHISAHRCAL
TOS8086	EALLGSKDGFLTKRIKSYETDRNDPTKPRALSGLSPYLHFGHISAHRCAL
Maro Goudo	EALLGSKDGFLTKRIKSYETDRNDPTKPRALSALSPYLHFGHISAQRCAL
TOS7940	EALLGSKDGFLTKRIKSYETDRNDPTKPRALSGLSPYLHFGHISAHRCAL
TOS10589	EALLGSKDGFLTKRIKSYETDRNDPTKPRALSGLSPYLHFGHISAHRCAL
TOG12380	EALLGSKDGFLTKRIKSYETDRNDPTKPRALSALSPYLHFGHISAQRCAL
TOG14928	EALLGSKDGFLTKRIKSYETDRNDPTKPRALSALSPYLHFGHISAQRCAL
MB3	EALLGSKDGFLTKRIKSYETDRNDPTKPRALSALSPYLHFGHISAQRCAL
TOB14466	EALLGSKDGFLTKRIKSYETDRNDPTKPRALSGLSPYLHFGHISAHRCAL
TOB7307	EALLGSKDGFLTKRIKSYETDRNDPTKPRALSALSPYLHFGHISAQRCAL

310 320 330 340 350

Sasanishiki	EAKKCRHLSPKSVDAFLEELVVRRELADNFCYYQPQYDSLSGAWEWARKT
Norin1	EAKKCRHLSPKSVDAFLEELVVRRELADNFCYYQPQYDSLSGAWEWARKT
Surjamkhi	EAKKCRHLSPKSVDAFLEELVVRRELADNFCYYQPQYDSLSGAWEWARKT
C7251	EAKKCRHLSPKSVDAFLEELVVRRELADNFCYYQPQYDSLSGAWEWARKT
Jiakawo Wodewo	EAKKCRHLSPKSVDAFLEELVVRRELADNFCYYQPQYDSLSGAWEWARKT
TOS13699	EAKKCRHLSPKSVDAFLEELVVRRELADNFCYYQPQYDSLSGAWEWARKT
TOS13649	EAKKCRHLSPKSVDAFLEELVVRRELADNFCYYQPQYDSLSGAWEWARKT
TOS8722	EAKKCRHLSPKSVDAFLEELVVRRELADNFCYYQPQYDSLSGAWEWARKT
TOS14844	EAKKCRHLSPKSVDAFLEELVVRRELADNFCYYQPQYDSLSGAWEWARKT
TOS8086	EAKKCRHLSPKSVDAFLEELVVRRELADNFCYYQPQYDSLSGAWEWARKT
Maro Goudo	EAKKCRHLSPKSVDAFLEELVVRRELADNFCYYQPQYDSLSGAWEWARKT
TOS7940	EAKKCRHLSPKSVDAFLEELVVRRELADNFCYYQPQYDSLSGAWEWARKT
TOS10589	EAKKCRHLSPKSVDAFLEELVVRRELADNFCYYQPQYDSLSGAWEWARKT
TOG12380	EAKKCRHLSPKSVDAFLEELVVRRELADNFCYYQPQYDSLSGAWEWARKT
TOG14928	EAKKCRHLSPKSVDAFLEELVVRRELADNFCYYQPQYDSLSGAWEWARKT
MB3	EAKKCRHLSPKSVDAFLEELVVRRELADNFCYYQPQYDSLSGAWEWARKT
TOB14466	EAKKCRHLSPKSVDAFLEELVVRRELADNFCYYQPQYDSLSGAWEWARKT
TOB7307	EAKKCRHLSPKSVDAFLEELVVRRELADNFCYYQPQYDSLSGAWEWARKT

360 370 380 390 400

Sasanishiki	LMDHAADKREHIYTREQLENAKTHDPLWNASQLEMVHHGKMHGFMRMYWA
Norin1	LMDHAADKREHIYTREQLENAKTHDPLWNASQLEMVHHGKMHGFMRMYWA
Surjamkhi	LMDHAADKREHIYTREQLENAKTHDPLWNASQLEMVHHGKMHGFMRMYWA
C7251	LMDHAADKREHIYTREQLENAKTHDPLWNASQLEMVHHGKMHGFMRMYWA
Jiakawo Wodewo	LMDHAADKREHIYTREQLENAKTHDPLWNASQLEMVHHGKMHGFMRMYWA
TOS13699	LMDHAADKREHIYTREQLENAKTHDPLWNASQLEMVHHGKMHGFMRMYWA
TOS13649	LMDHAADKREHIYTREQLENAKTHDPLWNASQLEMVHHGKMHGFMRMYWA
TOS8722	LMDHAADKREHIYTREQLENAKTHDPLWNASQLEMVHHGKMHGFMRMYWA
TOS14844	LMDHAADKREHIYTREQLENAKTHDPLWNASQLEMVHHGKMHGFMRMYWA
TOS8086	LMDHAADKREHIYTREQLENAKTHDPLWNASQLEMVHHGKMHGFMRMYWA
Maro Goudo	LMDHAADKREHIYTREQLENAKTHDPLWNASQLEMVHHGKMHGFMRMYWA
TOS7940	LMDHAADKREHIYTREQLENAKTHDPLWNASQLEMVHHGKMHGFMRMYWA
TOS10589	LMDHAADKREHIYTREQLENAKTHDPLWNASQLEMVHHGKMHGFMRMYWA
TOG12380	LMDHAADKREHIYTREQLENAKTHDPLWNASQLEMVHHGKMHGFMRMYWA
TOG14928	LMDHAADKREHIYTREQLENAKTHDPLWNASQLEMVHHGKMHGFMRMYWA
MB3	LMDHAADKREHIYTREQLENAKTHDPLWNASQLEMVHHGKMHGFMRMYWA
TOB14466	LMDHAADKREHIYTREQLENAKTHDPLWNASQLEMVHHGKMHGFMRMYWA
TOB7307	LMDHAADKREHIYTREQLENAKTHDPLWNASQLEMVHHGKMHGFMRMYWA

410 420 430 440 450

Sasanishiki	KKILEWTSGPEEALSTAIYLNDKYEIDGRDPSGYVGCMWSICGLHDQGWK
Norin1	KKILEWTSGPEEALSTAIYLNDKYEIDGRDPSGYVGCMWSICGLHDQGWK
Surjamkhi	KKILEWTSGPEEALSTAIYLNDKYEIDGRDPSGYVGCMWSICGLHDQGWK
C7251	KKILEWTSGPEEALSTAIYLNDKYEIDGRDPSGYVGCMWSICGLHDQGWK
Jiakawo Wodewo	KKILEWTSGPEEALSTAIYLNDKYEIDGRDPSGYVGCMWSICGLHDQGWK
TOS13699	KKILEWTSGPEEALSTAIYLNDKYEIDGRDPSGYVGCMWSICGLHDQGWK

TOS13649	KKILEWTSGPEEALSTAIYLNDKYEIDGRDPSGYVGCMWSICGLHDQGWK
TOS8722	KKILEWTSGPEEALSTAIYLNDKYEIDGRDPSGYVGCMWSICGLHDQGWK
TOS14844	KKILEWTSGPEEALSTAIYLNDKYEIDGRDPSGYVGCMWSICGLHDQGWK
TOS8086	KKILEWTSGPEEALSTAIYLNDKYEIDGRDPSGYVGCMWSICGLHDQGWK
Maro Goudo	KKILEWTSGPEEALSTAIYLNDKYEIDGRDPSGYVGCMWSICGLHDQGWK
TOS7940	KKILEWTSGPEEALSTAIYLNDKYEIDGRDPSGYVGCMWSICGLHDQGWK
TOS10589	KKILEWTSGPEEALSTAIYLNDKYEIDGRDPSGYVGCMWSICGLHDQGWK
TOG12380	KKILEWTSGPEEALSTAIYLNDKYEIDGRDPSGYVGCMWSICGLHDQGWK
TOG14928	KKILEWTSGPEEALSTAIYLNDKYEIDGRDPSGYVGCMWSICGLHDQGWK
MB3	KKILEWTSGPEEALSTAIYLNDKYEIDGRDPSGYVGCMWSICGLHDQGWK
TOB14466	KKILEWTSGPEEALSTAIYLNDKYEIDGRDPSGYVGCMWSICGLHDQGWK
TOB7307	KKILEWTSGPEEALSTAIYLNDKYEIDGRDPSGYVGCMWSICGLHDQGWK

	460 470 480 490 500
Sasanishiki	ERPVFGKIRYMNYAGCKRKFDVDAYISYVKRLAGQSKKRNAEESPNPVVK
Norin1	ERPVFGKIRYMNYAGCKRKFDVDAYISYVKRLAGQSKKRNAEESPNPVVK
Surjamkhi	ERPVFGKIRYMNYAGCKRKFDVDAYISYVKRLAGQSKKRNAEESPNPVVK
C7251	ERPVFGKIRYMNYAGCKRKFDVDAYISYVKRLAGQSKKRNAEESPNPVVK
Jiakawo Wodewo	ERPVFGKIRYMNYAGCKRKFDVDAYISYVKRLAGQSKKRNAEESPNPVVK
TOS13699	ERPVFGKIRYMNYAGCKRKFDVDAYISYVKRLAGQSKKRNAEESPNPVVK
TOS13649	ERPVFGKIRYMNYAGCKRKFDVDAYISYVKRLAGQSKKRNAEESPNPVVK
TOS8722	ERPVFGKIRYMNYAGCKRKFDVDAYISYVKRLAGQSKKRNAEESPNPVVK
TOS14844	ERPVFGKIRYMNYAGCKRKFDVDAYISYVKRLAGQSKKRNAEESPNPVVK
TOS8086	ERPVFGKIRYMNYAGCKRKFDVDAYISYVKRLAGQSKKRNAEESPNPVVK
Maro Goudo	ERPVFGKIRYMNYAGCKRKFDVDAYISYVKRLAGQSKKRNAEESPNPVVK
TOS7940	ERPVFGKIRYMNYAGCKRKFDVDAYISYVKRLAGQSKKRNAEESPNPVVK
TOS10589	ERPVFGKIRYMNYAGCKRKFDVDAYISYVKRLAGQSKKRNAEESPNPVVK
TOG12380	ERPVFGKIRYMNYAGCKRKFDVDAYISYVKRLAGQSKKRNAEESPNPVVK
TOG14928	ERPVFGKIRYMNYAGCKRKFDVDAYISYVKRLAGQSKKRNAEESPNPVVK
MB3	ERPVFGKIRYMNYAGCKRKFDVDAYISYVKRLAGQSKKRNAEESPNPVVK
TOB14466	ERPVFGKIRYMNYAGCKRKFDVDAYISYVKRLAGQSKKRNAEESPNPVVK
TOB7307	ERPVFGKIRYMNYAGCKRKFDVDAYISYVKRLAGQSKKRNAEESPNPVVK

Sasanishiki	LSKSQH
Norin1	LSKSQH
Surjamkhi	LSKSQH
C7251	LSKSQH
Jiakawo Wodewo	LSKSQH
TOS13699	LSKSQH
TOS13649	LSKSQH
TOS8722	LSKSQH
TOS14844	LSKSQH
TOS8086	LSKSQH
Maro Goudo	LSKSQH
TOS7940	LSKSQH
TOS10589	LSKSQH
TOG12380	LSKSQH
TOG14928	LSKSQH
MB3	LSKSQH
TOB14466	LSKSQH
TOB7307	LSKSQH

Figure S3. Amino acid sequences of the CPD photolyase of African rice species examined in this study in comparison with *O. sativa* Sasanishiki, Norin 1 and Surjamkhi. Red boxes indicate amino acid changes in comparison with Sasanishiki.

Supplementary Figure S4

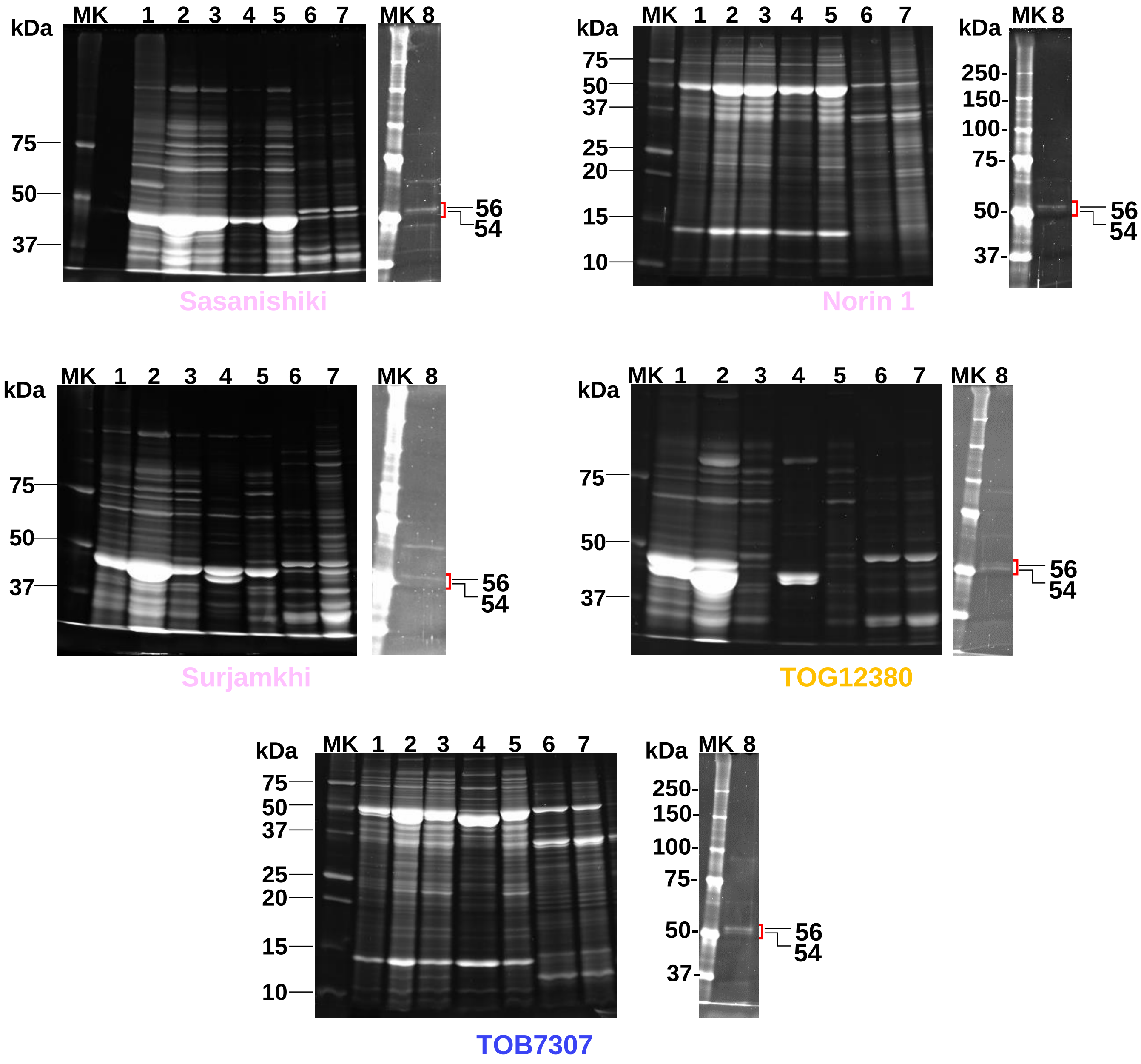

Figure S4. Purification of native CPD photolyase from rice plant. Lane 1, crude extract (fraction 1); lane 2, protein precipitated with ammonium sulphate between 35% and 70% saturation (fraction 2); lane 3, flow-through from a UNO-Q12 anion-exchange column (fraction 3); lane 4, protein eluted from the UNO-Q12 column; lane 5, flow-through from a heparin affinity column; lane 6, protein eluted from the heparin column (fraction 4); lane 7, protein not bound to UV-irradiated DNA-conjugated magnetic beads; lane 8, protein purified with UV-irradiated DNA-conjugated magnetic beads (fraction 5). Sasanishiki, Norin 1 and Surjamkhi are Asian *O. sativa*; TOG12380 is *O. glaberrima* and TOB7307 is *O. barthii*. Electrophoresis was performed in a 12.5% (Norin 1 and TOB7307) or 7.5% [Sasanishiki, Surjamkhi and TOG12380 and all lane 8 (fraction 5)] SDS-polyacrylamide gel; the gels were stained with SYPRO Ruby stain. MK, molecular weight marker. Outline red colour indicated phosphorylated (56 kDa) and unphosphorylated (54 kDa) purified CPD photolyase protein.

Supplementary Figure S5

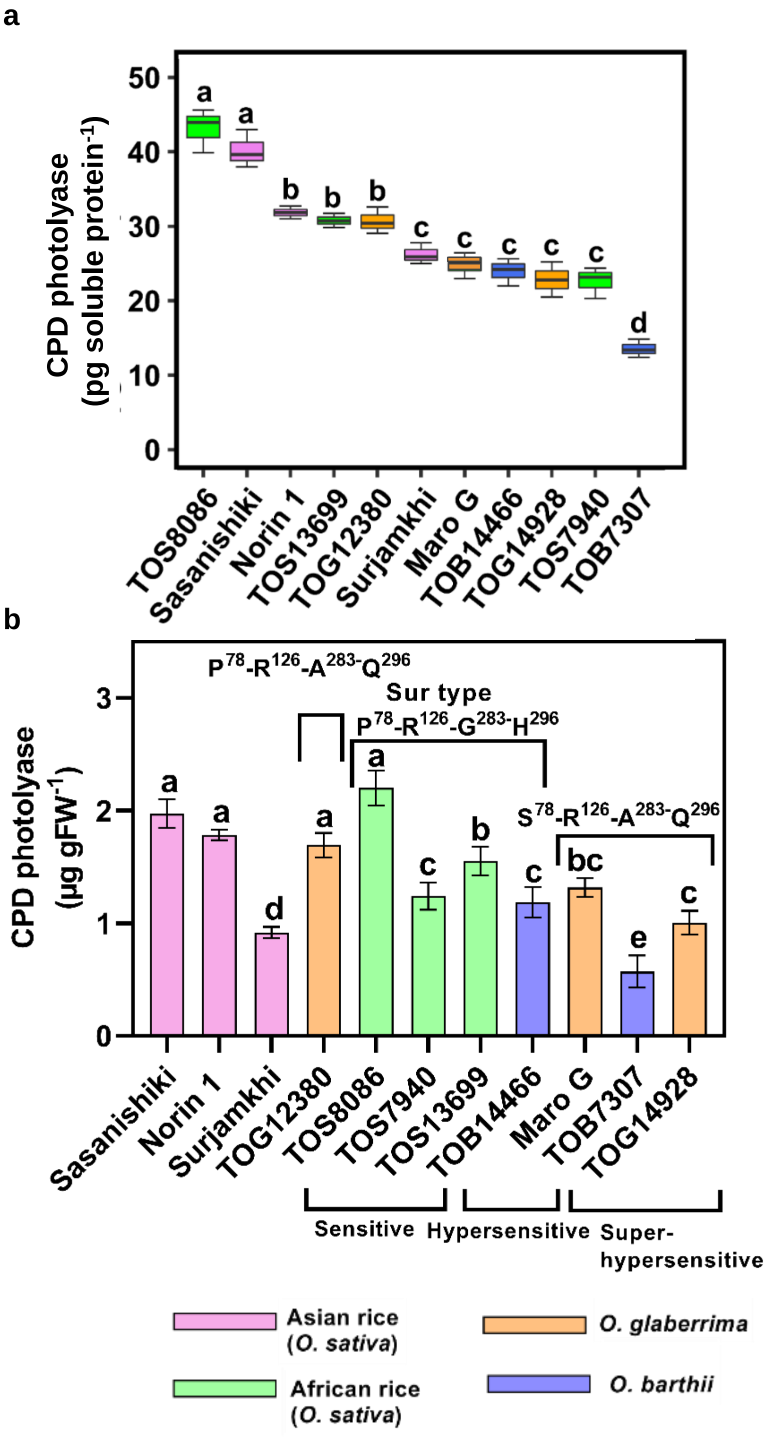

Figure S5. CPD photolyase content in crude extracts. a, CPD photolyase content was obtained by dividing CPD photorepair activity of the purified CPD photolyase (CPD Mb^{-1} min^{-1} ng of purified $protein^{-1}$) by that in crude extracts (CPD Mb^{-1} min^{-1} μg of soluble $protein^{-1}$). Box plots show median as the central measure and 25th percentile and 75th percentile. **b**, UVB-super-hypersensitive African rice species have low content of the CPD photolyase protein. The amount of CPD photolyase (gFW^{-1}) was calculated by dividing total activity of CPD photolyase by CPD photorepair activity of purified CPD photolyase. Values are the mean ± SD. n=3 repeats; different letters above the boxplots and bar graph indicate significant differences determined by the Tukey-Kramer test ($P < 0.05$).

Supplementary Figure S6

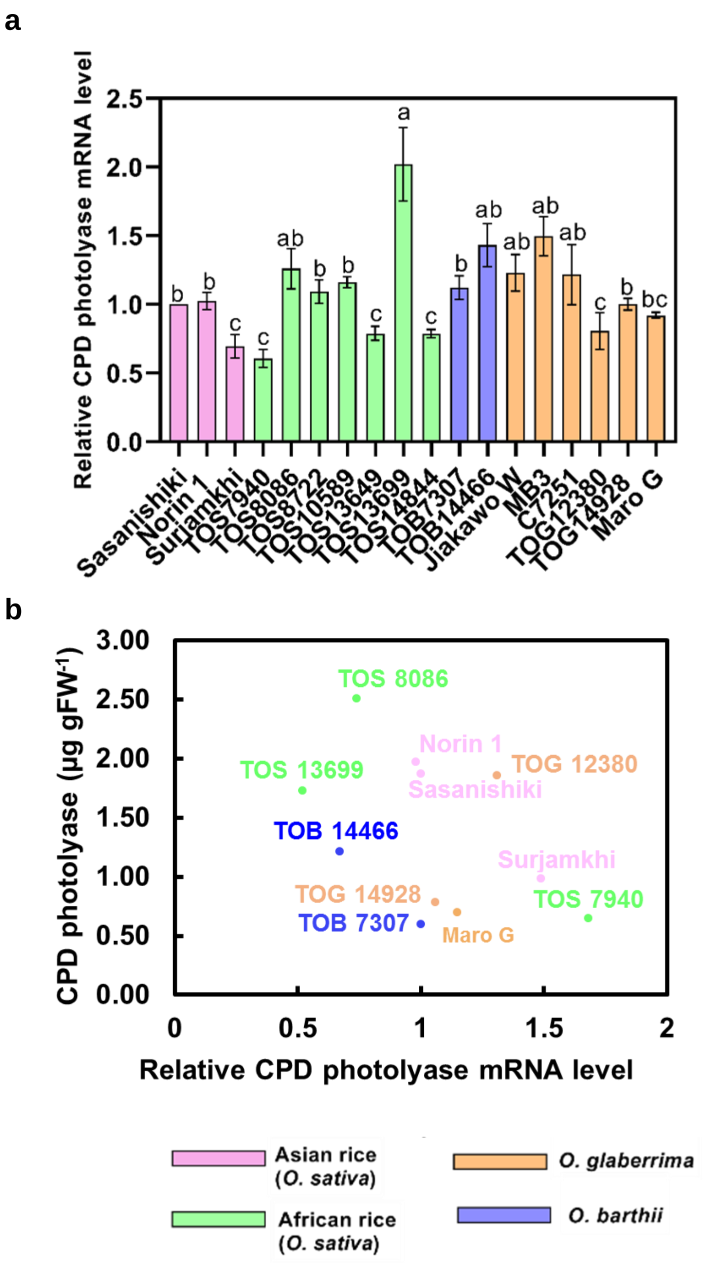

Figure S6. Relationship between CPD photolyase mRNA abundance and CPD photolyase content per gram fresh weight. a, mRNA abundance in third fully expanded leaves was measured by quantitative real-time RT-PCR. All data were normalized to the level of actin mRNA and are shown relative to the mRNA level in Sasanishiki. Values are the mean ± SD. n=3 repeats; different letters indicate significant differences determined by the Tukey-Kramer test ($P < 0.05$) . **b** , Relationship between CPD photolyase mRNA level in **a** and CPD photolyase content (data from Supplementary Fig. S5b). There was no significant correlation between the data sets.

Supplementary Figure S7

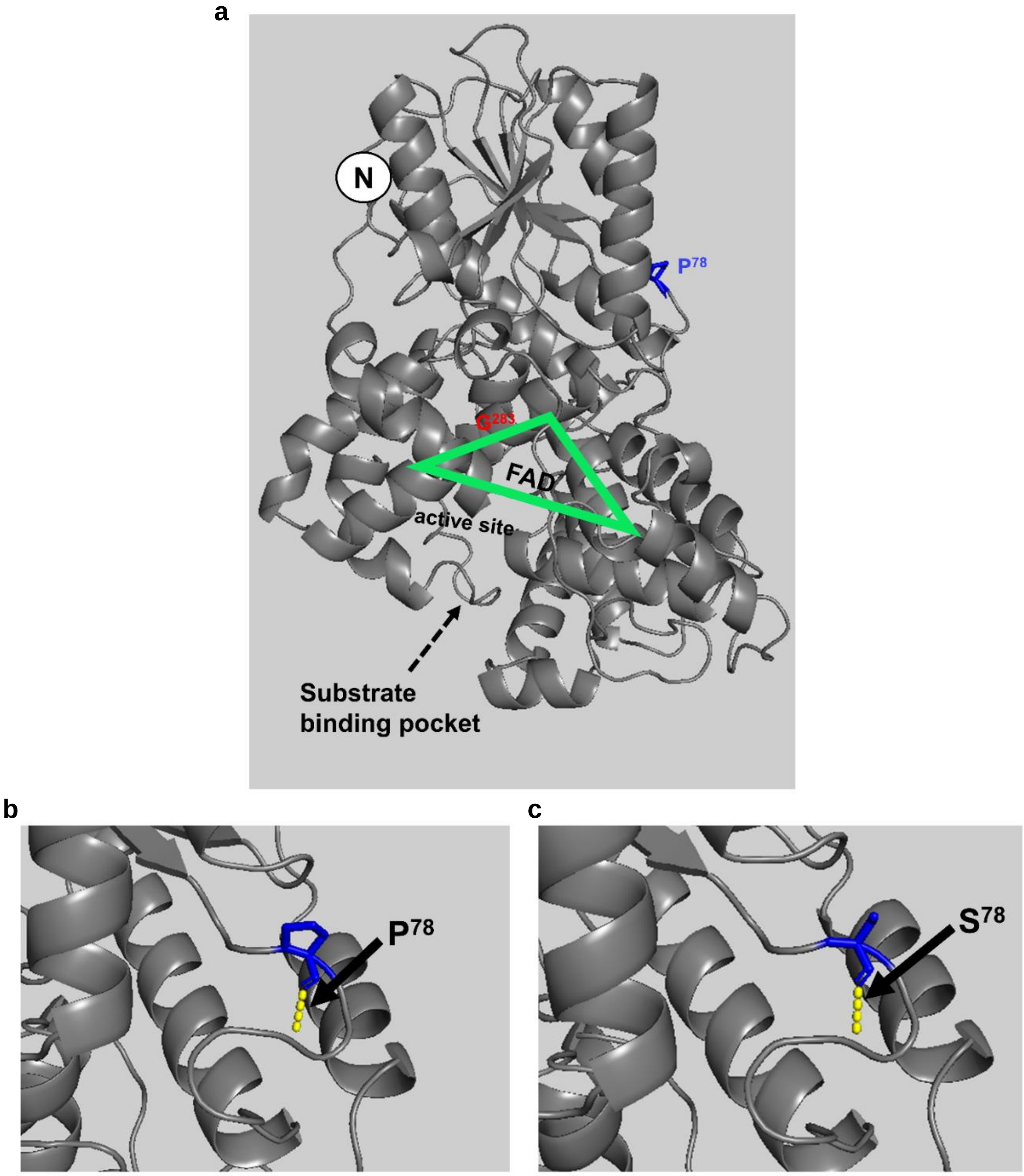

Figure S7. 3D structure of Sasa-type CPD photolyase and TOB7307. Visualization of rice CPD photolyase 3D structure was analysed by PyMol 2.2.2 software using amino sequence information of selected cultivar TOB7307 compared to that of Sasanishiki (protein data bank code: 3UMV)[23]. **a**, 3D structure of Sasanishiki rice CPD photolyase, green triangle indicates the position of FAD chromophores, blue colour indicated amino acid position 78, red colour indicated amino acids position 283 and black dotted arrow indicate catalytic site of the enzyme. **b** and **c**, 3D structure of Sasa-type CPD photolyase containing Proline (P) at amino acid 78 (**b**), and TOB7307 CPD photolyase containg Serine (S) at amino acid 78 (**c**). Change of P^{78} to S in cultivar TOB7307 did not cause a change of 3D structure. N = N terminal. Yellow dotted lines indicated polar contact of amino acid position 78.

a

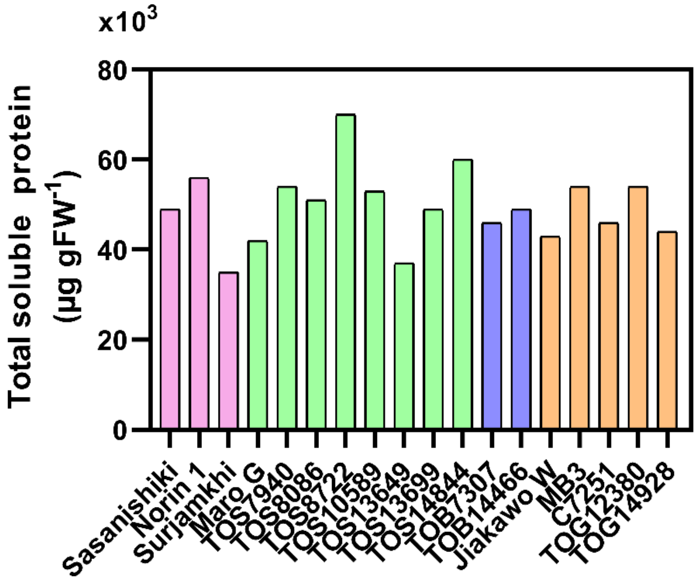

b

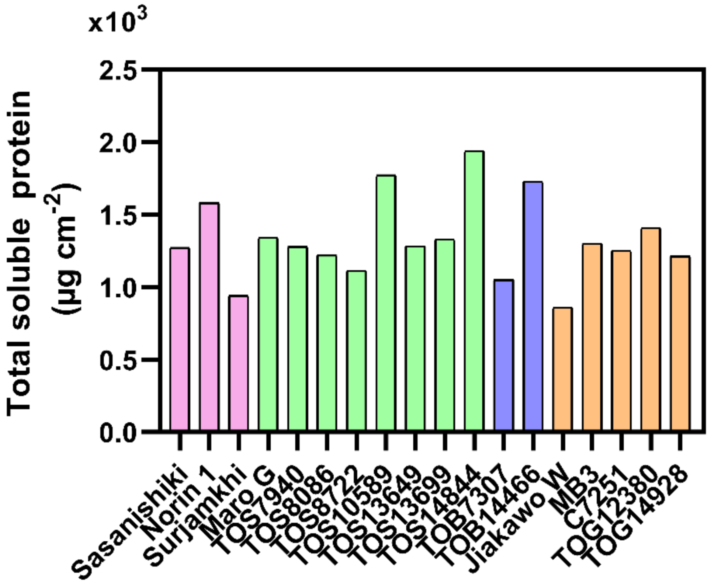

Figure S8. Total soluble protein content of third fully expanded leaves. Total soluble protein content per gram fresh weight (gFW) (**a**) or per area (cm^2) (**b**) of third fully expanded leaves. Protein amount was measured by the Bradford assay.

Supplementary Figure S9

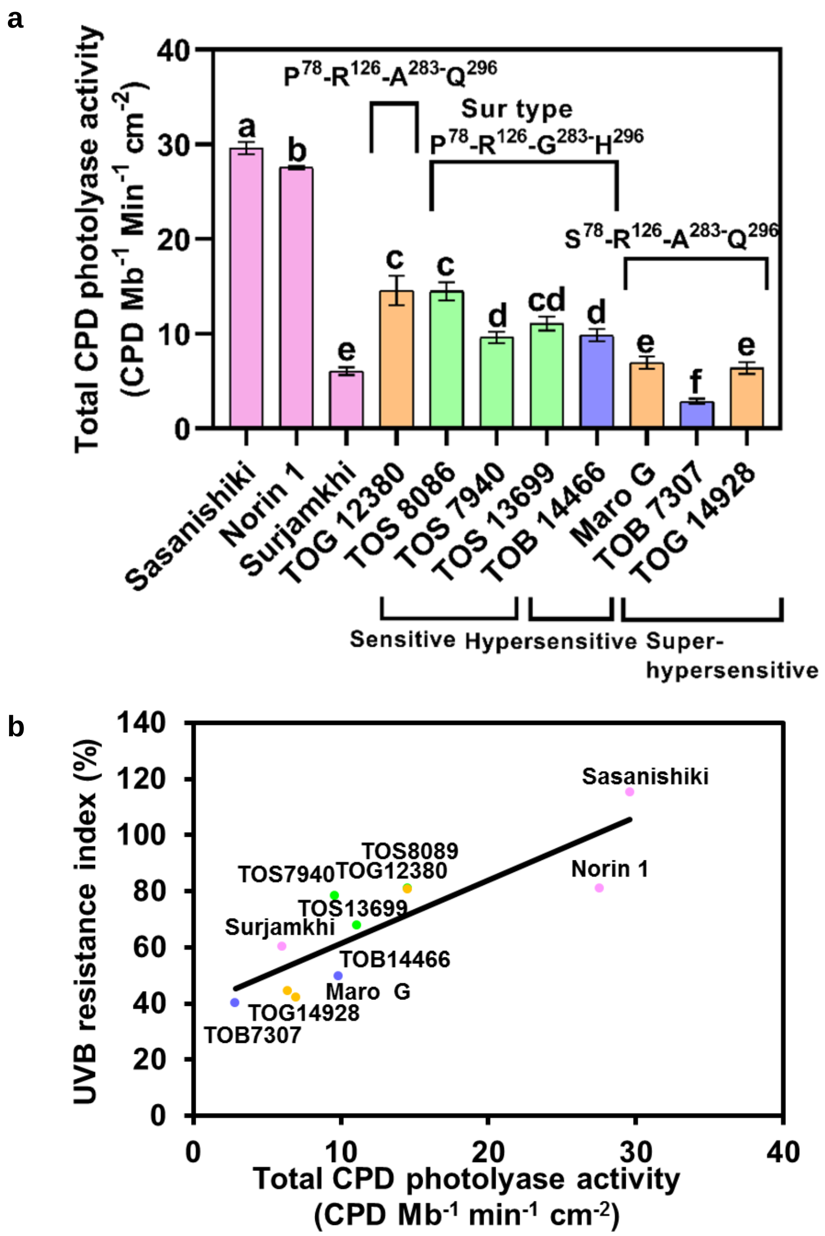

Figure S9. UVB resistance index is strongly correlated with total activity of CPD photolyase in the leaf. a, Total CPD photolyase activity was calculated from CPD photolyase protein content [(pg of CPD photolyase) (µg of soluble protein)$^{-1}$], total soluble protein content per leaf area (µg cm^{-2}) and activity of CPD photolyase (CPD Mb^{-1} min^{-1} ng^{-1}). UVB-super-hypersensitive African rice cultivars have low total activity of CPD photolyase. **b**, Correlation between UVB resistance index (Fig. 1d) and total CPD photolyase activity shown in (**a**). Linear regression analysis showed significant correlation between the data sets ($P < 0.01$; $R^2 = 0.72$). Values are means ± SD. n=3 repeats in **b**; different letters indicate significant differences determined by the Tukey-Kramer test ($P < 0.05$). Colour coding is as in Fig. 1.

Supplementary Figure S10

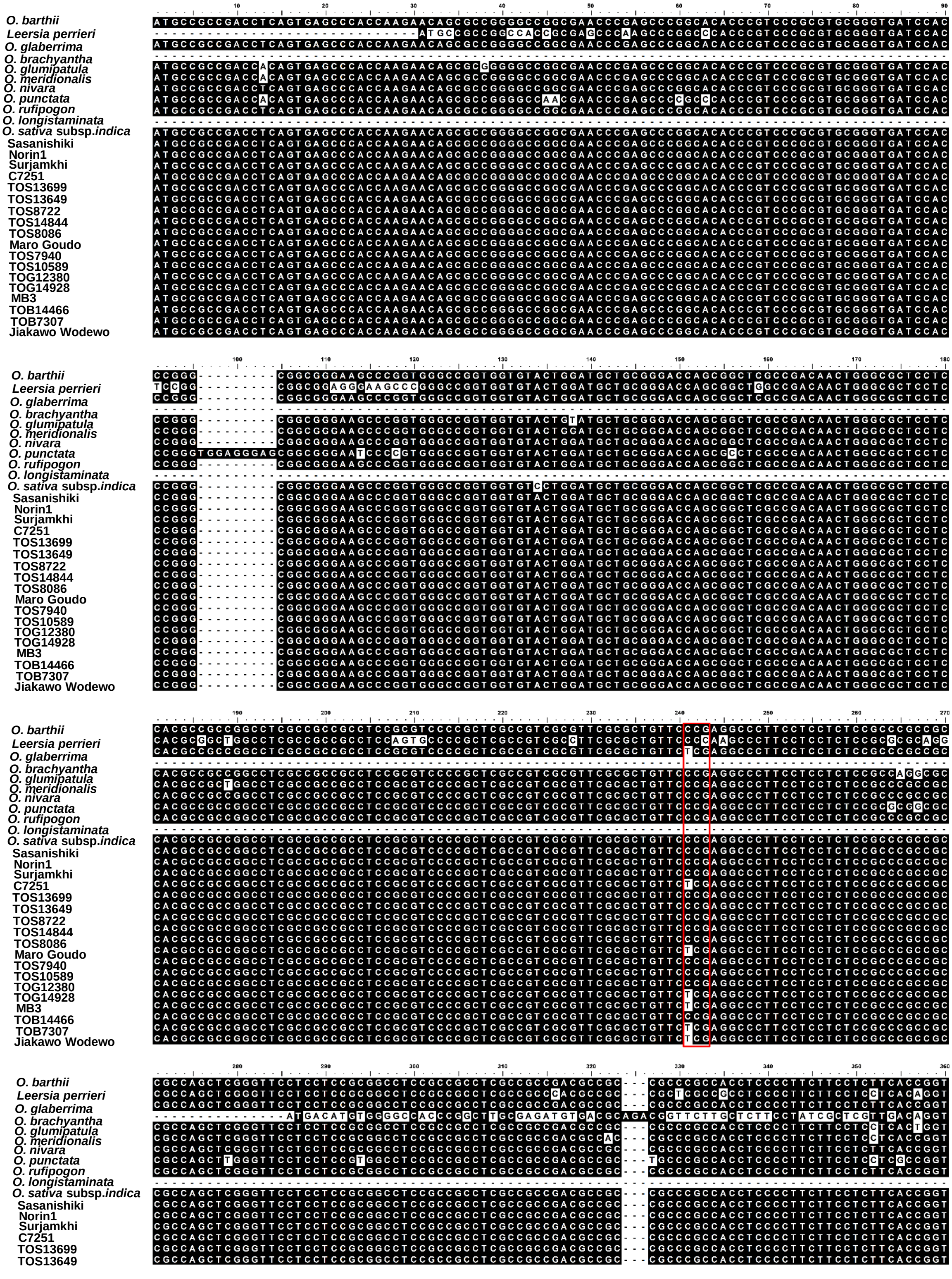

Supplementary Figure S10 continued

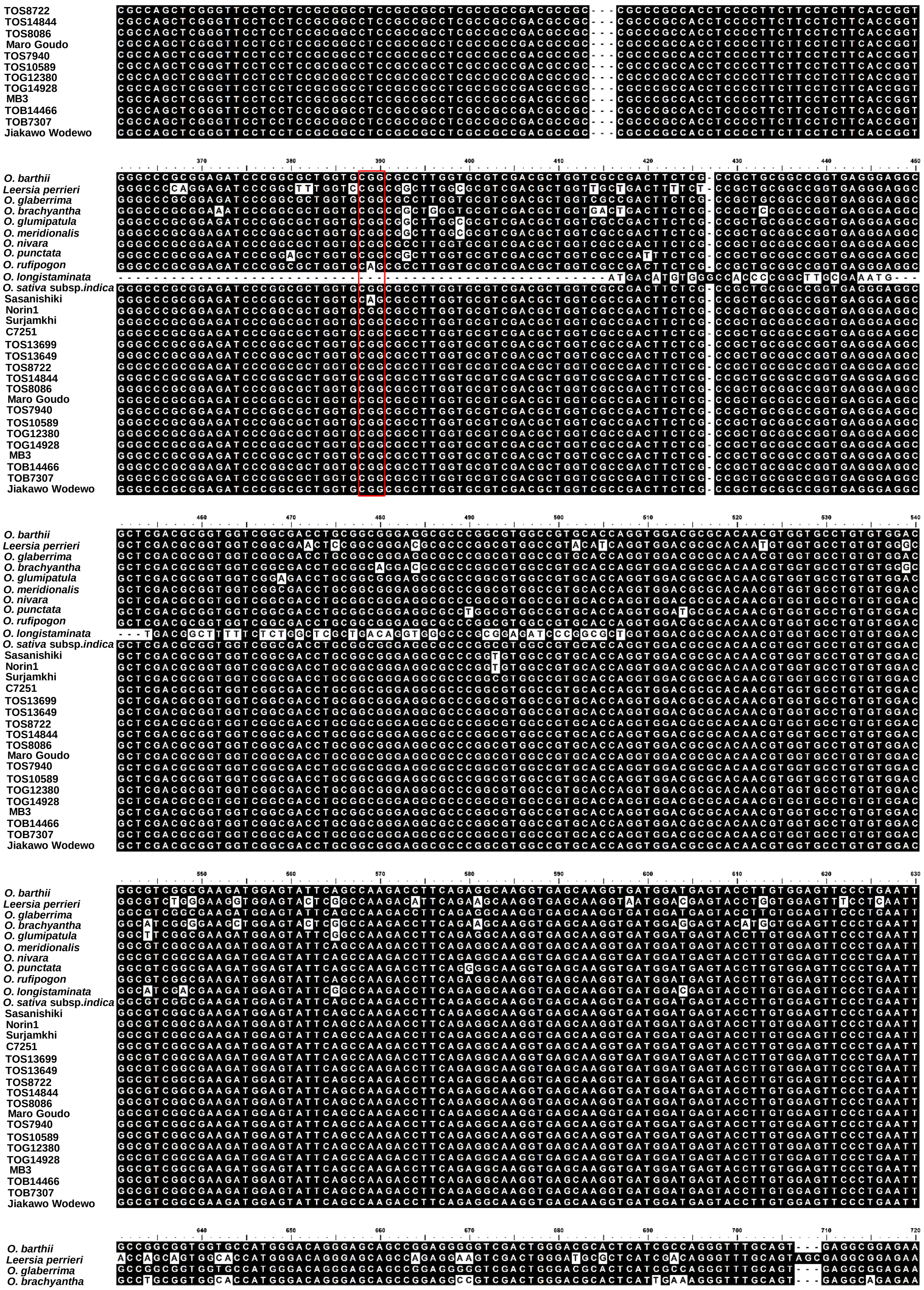

Supplementary Figure S10 continued

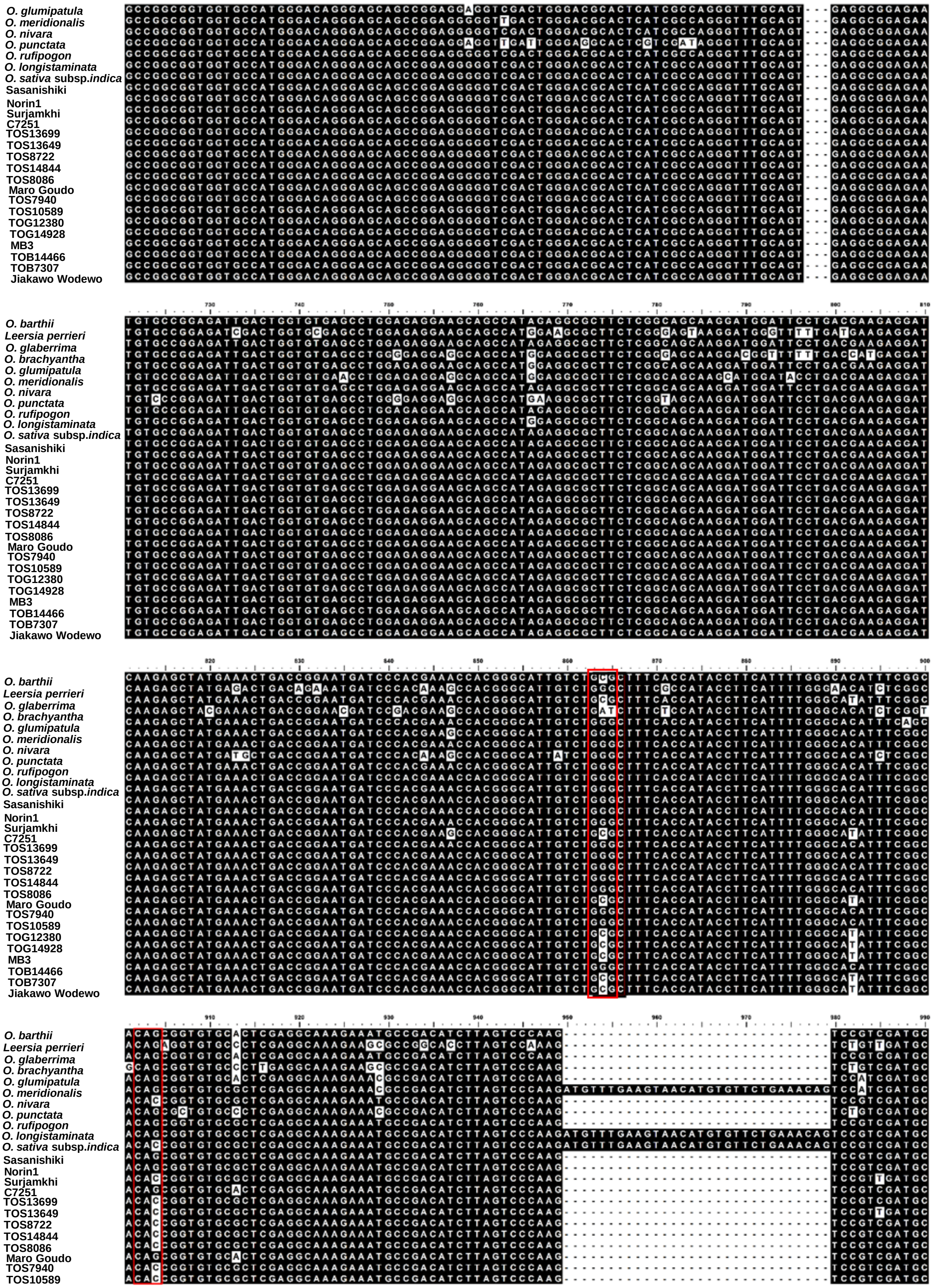

Supplementary Figure S10 continued

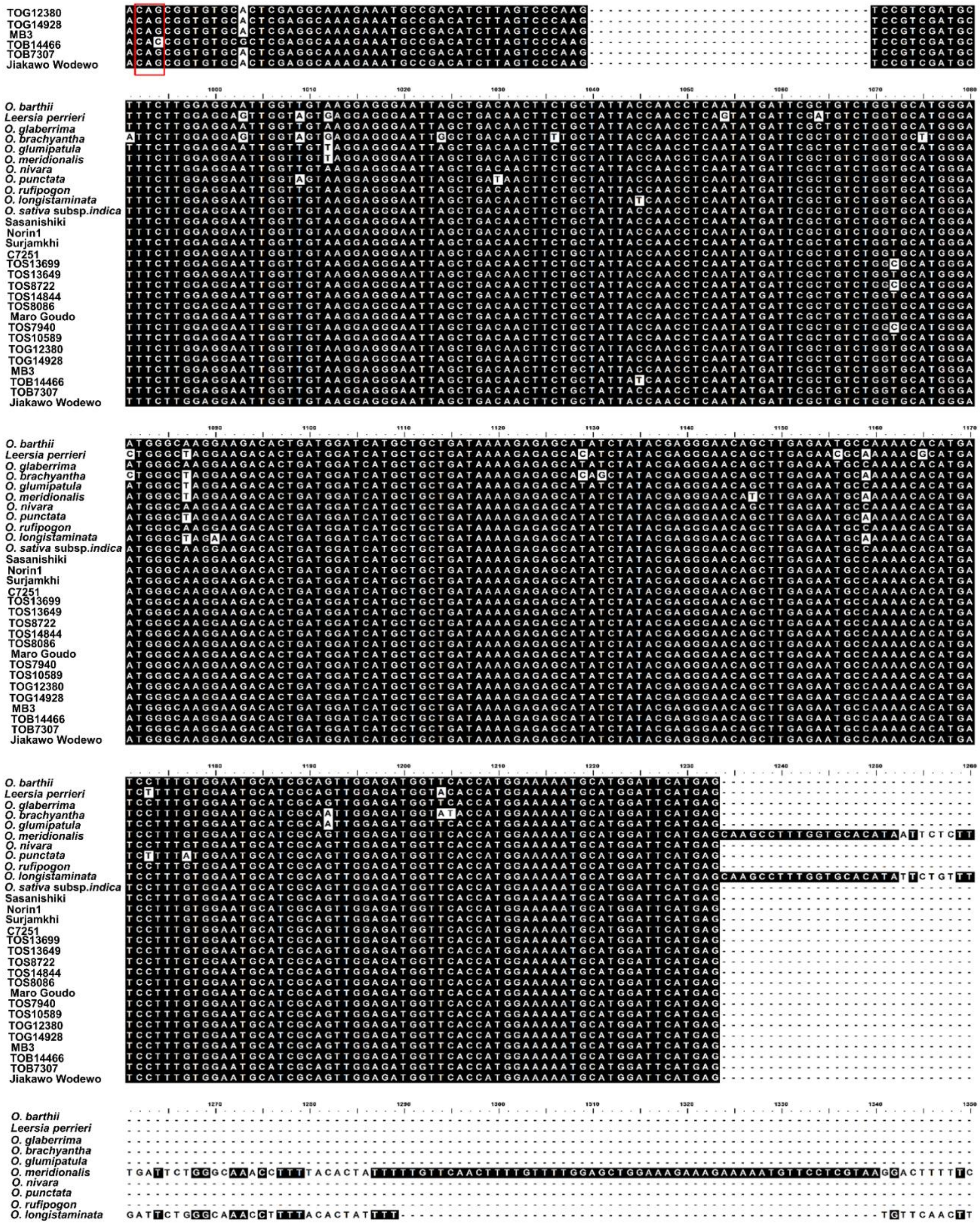

Figure S10. Genomic sequences of CPD photolyases of wild rice species and Asian and African rice cultivars. Wild rice species: *O. brachyantha*, *O. punctata*, *O. glumipatula*, *O. meridionalis*, *O. longistaminata*, *O. rufipogon*, *O. nivara* and *O. barthii*; Asian rice cultivars: Sasanishiki, Norin 1 and Surjamkhi; African rice cultivars: *O. sativa* (TOS) 7940, 8086, 8722, 10589, 13649, 13699 and 14844, tropical *O. barthii* (TOB) 7307 and 14466; tropical *O. glaberrima* (TOG) 12380, 14928, Jiakawo Wodewo, MB3, C7251 and Maro Goudo. Red boxes indicate nucleotide changes at positions 232, 377, 848 and 888 of African rice cultivars in comparison with both wild and Asian rice. *Leersia perrieri* was used as an outgroup species.

Supplementary Figure S11

Position of amino acid	78	126	283	296
*Leersia perrieri**	P	R	G	Q
O. brachyantha	P	R	D	Q
O. punctata	P	R	G	Q
O. meridionalis	P	R	G	Q
O. longistaminata	-	-	G	Q
O. rufipogon	P	R	G	H
O. nivara	P	R	G	H
Sasanishiki	P	Q	G	Q
Norin 1	P	R	G	Q
Surjamkhi	P	R	G	H
O. barthii	P	R	A	Q
O. glaberrima	S	R	A	
Maro Goudo	S	R	A	Q
TOB7307	S	R	A	Q
Jiakawo Wodewo	S	R	A	Q
TOG12380	P	R	A	Q
TOG14928	S	R	A	Q

* Outgroup species *L. perrieri*

Wild rice species

Asian rice

African rice (*O. sativa*)

African rice (*O. barthii*)

African rice (*O. glaberrima*)

Figure S11. Amino acid sequences of CPD photolyases of wild rice species and Asian and African rice cultivars. Wild rice species: *O. brachyantha*, *O. punctata*, *O. glumipatula*, *O. meridionalis*, *O. longistaminata*, *O. rufipogon*, *O. nivara* and *O. barthii*; Asian rice cultivars: Sasanishiki, Norin 1 and Surjamkhi; African rice cultivars: TOB7307 (*O. barthii*) and tropical *O. glaberrima* (TOG) 12380, 14928, Jiakawo Wodewo. Red box indicates amino acids at positions 78 and 283 of African rice cultivars in comparison with both wild and Asian rice.

Table S1 The effect of UVB radiation on tiller number and fresh weight of the above-ground parts of rice and UVB resistance index.

		Cultivar	Tiller number			Weight (g)			UVB-resistance index [c]
			-UVB	+UVB	Ratio[a]	-UVB	+UVB	Ratio[b]	
Asian rice	*O. sativa*	Sasanishiki	3.3±0.6[d]	2.2±0.3	0.67	1.27±0.30	0.63±0.15	0.50	117
		Norin 1	3.3±0.9	1.6±0.2	0.48	1.57±0.31	0.53±0.07	0.34	82
		Surjamkhi	3.0±0.4	1.2±1.2	0.40	1.73±0.16	0.35±0.05	0.20	60
African rice	*O. sativa*	TOS7940	3.6±0.6	1.9±0.2	0.53	1.87±0.24	0.47±0.08	0.25	78
		TOS8086	2.6±0.3	1.5±0.2	0.58	2.46±0.25	0.68±0.04	0.28	86
		TOS8722	3.0±0.0	1.0±0.0	0.33	1.66±0.10	0.32±0.03	0.19	52
		TOS10589	3.0±0.9	1.1±0.2	0.37	2.34±0.24	0.50±0.05	0.21	58
		TOS13649	2.9±0.1	1.5±0.0	0.52	1.24±0.06	0.49±0.02	0.40	92
		TOS13699	2.8±0.3	1.1±0.1	0.39	2.26±0.10	0.63±0.07	0.28	67
		TOS14844	2.4±0.3	1.0±0.0	0.42	2.88±0.22	0.75±0.08	0.26	68
	O. barthii	TOB7307	2.9±0.1	1.0±0.0	0.34	2.28±0.35	0.13±0.04	0.06	40
		TOB14466	3.4±1.1	1.1±0.1	0.32	2.61±0.26	0.50±0.09	0.19	51
	O. glaberrima	TOG12380	3.8±0.8	2.0±0.1	0.53	3.14±0.32	0.86±0.11	0.27	80
		TOG14928	2.8±0.3	1.0±0.0	0.36	2.79±0.01	0.22±0.03	0.08	44
		Jiakawo Wodewo	3.1±0.5	1.0±0.0	0.32	1.81±0.14	0.23±0.01	0.13	45
		MB3	2.8±0.3	1.0±0.0	0.36	2.50±0.34	0.55±0.13	0.22	58
		C7251	3.1±0.3	1.0±0.0	0.32	2.46±0.15	0.25±0.06	0.10	42
		Maro Goudo	3.0±0.6	1.0±0.0	0.33	2.29±0.14	0.19±0.05	0.08	41

[a]Ratio of irradiated to unirradiated tiller number = (+UVB)/Control (-UVB)
[b]Ration of irradiated to unirradiated fresh weight = (+UVB)/Control (-UVB)
[c]Resistance index was determined by summing the value of ratio of irradiated to unirradiated tiller number and fresh weight; Resistance index = (a+b) x100
[d]Standard deviation, *n*=9

Supplementary Table S2

Table S2 Purification fold of native CPD photolyase.

	In vitro CPD photolyase activity (CPD Mb^{-1} min^{-1} μg of crude extract $^{-1}$)	Activity of CPD photolyase (CPD Mb^{-1} min^{-1} μg of purified native CPD $photolyase^{-1}$)[a]	Purification fold[b]
Sasanishiki	0.0076	540	71053
Norin 1	0.0058	498	85862
Surjamkhi	0.0027	260	96296
TOS7940	0.0022	250	113636
TOG12380	0.0050	265	53000
TOB7370	0.0014	231	165000

[a] Protein amount was estimated from band intensity in SDS-PAGE gels.
[b] Purification fold was calculated by dividing CPD photolyase activity *in vitro* by activity of purified CPD photolyase.
